# Synthetic biosensors for precise gene control and real-time monitoring of metabolites

**DOI:** 10.1093/nar/gkv616

**Published:** 2015-07-07

**Authors:** Jameson K. Rogers, Christopher D. Guzman, Noah D. Taylor, Srivatsan Raman, Kelley Anderson, George M. Church

**Affiliations:** 1 Wyss Institute for Biologically Inspired Engineering, Harvard University, 3 Blackfan Circle, Boston, MA 02115, USA; 2 School of Engineering and Applied Sciences, Harvard University, 29 Oxford Street, Cambridge, MA 02143, USA; 3 Department of Genetics, Harvard Medical School, 77 Avenue Louis Pasteur, Boston, MA 02115, USA

## Abstract

Characterization and standardization of inducible transcriptional regulators has transformed how scientists approach biology by allowing precise and tunable control of gene expression. Despite their utility, only a handful of well-characterized regulators exist, limiting the complexity of engineered biological systems. We apply a characterization pipeline to four genetically encoded sensors that respond to acrylate, glucarate, erythromycin and naringenin. We evaluate how the concentration of the inducing chemical relates to protein expression, how the extent of induction affects protein expression kinetics, and how the activation behavior of single cells relates to ensemble measurements. We show that activation of each sensor is orthogonal to the other sensors, and to other common inducible systems. We demonstrate independent control of three fluorescent proteins in a single cell, chemically defining eight unique transcriptional states. To demonstrate biosensor utility in metabolic engineering, we apply the glucarate biosensor to monitor product formation in a heterologous glucarate biosynthesis pathway and identify superior enzyme variants. Doubling the number of well-characterized inducible systems makes more complex synthetic biological circuits accessible. Characterizing sensors that transduce the intracellular concentration of valuable metabolites into fluorescent readouts enables high-throughput screening of biological catalysts and alleviates the primary bottleneck of the metabolic engineering design-build-test cycle.

## INTRODUCTION

In-depth biological part characterization forms the foundation for abstraction and complexity in engineered biological systems. Sensors are one of the most important components to characterize as they provide the channels of communication into and out of the cell. Biosensors that respond to external agents such as chemicals or light allow real-time control of gene expression. Furthermore, sensors enable online monitoring of metabolic phenotypes by transducing intracellular chemical concentration into gene expression. Because phenotype evaluation is a major rate-limiting step in metabolic engineering, coupling sensors to reporter gene expression enables rapid and multiplexed phenotype evaluation, facilitating faster design-build-test cycles.

Small molecule inducible systems are genetically encoded biosensors that modulate gene expression in response to the presence of a small molecule inducer. One of the most widely used biosensors is the allosteric DNA binding protein LacI, which natively regulates the lactose catabolism operon in *Escherichia coli* by binding near the transcriptional start site and repressing transcription initiation (1). When an inducing molecule such as isopropyl β-d-1-thiogalactopyranoside (IPTG) is present in the cell, it binds to the LacI protein and the LacI-IPTG complex disassociates from DNA, allowing transcription to proceed. Construction and characterization of engineered LacI-inducible systems (2,3) has resulted in widespread use in applications ranging from protein over-expression (4), to signal processing (5,6), and even chromosomal visualization (7).

Because of their general applicability and extensive characterization, a small set of canonical inducible regulators (LacI, TetR (2), AraC (8), LuxR (9)) are repeatedly used for a diverse range of applications. Other well-characterized inducible systems are available (PrpR (10), RhaRS (11), CymR (12), XylS (13)), but with the exception of CymR, these suffer from catabolite repression and/or weak induction. Other expression control paradigms include riboswitches (14), which provide ligand-mediated control of translation, and light-regulated optogenetic systems (15), which are a promising complement to chemical induction. However, there is a pressing need for additional inducible systems, as genetically encoded biosensors allow facile control of transcription by merely supplying the inducer in the growth medium.

Robust inducible systems are valuable tools facilitating adjustable and on-the-fly control of specific genes. Tunable expression of one or more genes over the course of an organism's growth provides unique insights into gene function (16) and developmental programs (17). Dynamic regulation is therefore distinct from static methods that disrupt genes altogether (18–20) or that change expression through modification of *cis*-regulatory elements such as promoters (21) and ribosomal binding sites (RBS) (22). Because extensive characterization is often lacking, inducible systems are typically operated as all-or-nothing switches without regard for the speed or extent of induction. This mode of operation is adequate for conditional overexpression of potentially toxic genes, but yields less information than careful titration of gene dosage when probing cellular behavior (23).

When metabolite-responsive biosensors regulate fluorescent reporters, they facilitate real-time observation of internal cell states. Measurement of intracellular metabolites is often desirable, but is typically a slow and destructive process. Standard methods require macroscopic cultures or prepared lysates, which are assayed by chromatography and mass spectrometry, or by absorbance in special cases (24). When intracellular metabolite concentration is transduced to fluorescence, high-throughput single cell measurements become possible (24). Fluorescent monitoring approaches can leverage allosteric transcriptional regulators that are already known for many common intermediates of metabolism, such as pyruvate (25), phosphoenol pyruvate (26), citrate (27), lactate (28), unsaturated fatty acids (29) and NADH (30).

Genetically encoded biosensors are valuable in metabolic engineering applications as they enable cells to report on their individual progress in producing a target compound from glucose or other low cost starting materials. Each cell expresses a fluorescent protein or antibiotic resistance gene at a rate proportional to its ability to produce the target compound. This link between internal metabolite concentration and reporter expression allows engineers to screen (by fluorescence) or select (with an antibiotic) for the most desirable cells (31). Coupling selections or screens to small molecule sensors have yielded new enzymes (32–35) and genomes enhanced for target metabolite production (36).

In addition to online observation of key metabolites, metabolic engineers benefit from advances in real-time control of biosynthetic gene expression. Independent control of each enzyme in a metabolic pathway facilitates the careful balancing of expression that is often necessary for optimal product production (37). In other cases, carefully timed expression of pathway enzymes has been shown to increase product titer (38). Keasling and coworkers used a genetically encoded biosensor to increase biodiesel titers by tying enzyme expression to the concentration of a pathway intermediate (39). Monitoring unwanted side-products, such as lactate, or desired products, such as fatty acids, with fluorescent reporters allows screening of millions of cells by flow-cytometry (32). If the reporter is an antibiotic resistance marker, selection can then be used in directed evolution applications (40).

Thorough characterization of an inducible system should provide enough information for applications ranging from environmental sensing and signal processing to metabolic engineering. Many characterization approaches provide this information and new methodologies are being developed. The BioFab has created analysis pipelines resulting in the characterization of transcriptional initiators (41) and terminators (42). Furthermore, they have developed methods to indicate how robust these characterized parts are to changing environments (43). While these characterization projects rely primarily on end-point measurements in 96-well plates, other approaches have exclusively used flow-cytometry data to fit parameters to complex models of induction (44).

Biosensor parameters dictate how useful a given biosensor will be for the application at hand. Characterizing these properties requires measuring: (1) the relationship between stimulus strength and circuit activation; (2) the response time of the biosensor to a stimulus; (3) the heterogeneity of biosensor activation between cells in an isogenic population and (4) the cross-reactivity with stimuli of other biosensors. Pioneering work from the Endy lab has introduced the idea of biological part ‘datasheets’ that capture the information subsequent engineers might need in order to use a new part (9). This engineering approach to biology has subsequently been used to characterize the commonly used inducible systems XylS, LacI and AraC (45). In this study, we characterize four additional allosteric transcription factors that modulate transcription in response to small molecule concentration. We include two commonly used biosensors in our analysis in order to ground our results in a context that many bioengineers are familiar with.

## MATERIALS AND METHODS

### Chemicals and reagents

All reagents were obtained from Sigma (St. Louis, MO, USA) unless otherwise noted. Antibiotics and IPTG were obtained from Gold Biotechnology (St. Louis, MO, USA). Anhydrotetracycline (aTC) was obtained from Clontech (Mountain View, CA, USA). Polymerase chain reaction (PCR) mix was purchased from Kapa Biosystems (Wilmington, MA, USA). Erythromycin and aTC were dissolved in ethanol while naringenin was dissolved in dimethyl sulfoxide. All other inducers were dissolved in deionized water.

### Plasmid construction

Plasmids were constructed using Gibson isothermal assembly methods (46) and transformed into DH5α electrocompetent cells (New England Biolabs, Ipswich, MA, USA). All standard induction plasmids contained the *rrnB* strong terminator (47) followed by the inducible promoter and the strong g10 RBS (48) ‘tttaactttaagaaggagatatacat,’ driving the expression of sfGFP (49) (except in the case of CdaR which used the native RBS). GFP was followed by a transcriptional terminator prefixing the proB promoter (50) and strong RBS ‘gaaataaggaggtaatacaa,’ which facilitated expression of the transcriptional regulator. Each inducible system was implemented on high and low copy plasmids. High copy pJKR-H plasmids were constructed with the pUC origin and beta lactamase antibiotic resistance marker derived from pUC19 (New England Biolabs, Ipswich, MA, USA). Low copy pJKR-L plasmids were constructed in the same way, except that the pUC origin was replaced by the SC101 origin (including repA) from pSC101 obtained from American Type Culture Collection (ATCC #37032). In the case of the MphR inducible system, the *eryR* erythromycin resistance cassette was included as well. The sequences of the transcriptional regulators and their cognate promoters are supplied in Supplementary Table S1. The plasmids MphR-p15a-SPEC-mCherry and AcuR-colA-KAN-CFP were designed for compatible maintenance with pJKR-H-CdaR. In both these plasmids the antibiotic resistance gene and origin of replication were replaced with p15a-*aadA* and colA-*kanR*. Sequence and organism names for each of the MIOX enzymes can be found in Supplementary Table S2. Each enzyme was cloned downstream of the constitutive promoter P2 (41) and g10 RBS to create pJKR-MIOX variants. These expression plasmids used the colA origin of replication and a kanamycin resistance gene for maintenance. The Udh enzyme was expressed constitutively on a p15a origin of replication providing spectinomycin resistance. Sequences and plasmids are available on Addgene (plasmid numbers 62557–62570).

### Induction and toxicity

DH5α cells transformed with pJKR plasmids and maintained with carbenicillin were used in the induction assays. For each induction evaluation experiment, the cells were grown overnight to saturation before being diluted 1:100 into fresh LB media and incubated at 200 RPM and 37°C. After 4 h, 150 μl of the log-phase cells were transferred to 96-well plates and stock inducer was added to achieve the desired range of induction concentrations. Three separate wells were inoculated and independently supplemented with the appropriate amount of inducing chemical for each level of induction. Measurements were made on the same Biotek (Winooksi, VT, USA) HT plate reader using the same settings: excitation 485/20, emission 528/20, 37°C and fast shaking. Fluorescence and absorbance were measured every 10 min for 15 h. Fluorescence was measured in arbitrary units (AFU) while optical density was determined by absorbance (OD). Normalized fluorescence was determined by dividing fluorescence by optical density for a given measurement. Five independent wells containing control strains, transformed with pUC19, were included to provide a measurement of background autofluorescence. The same protocol modified to observe fluorescence after 90 min was used to evaluate induction of the TtgR biosensor with phenol and related compounds.

**Figure 1.**
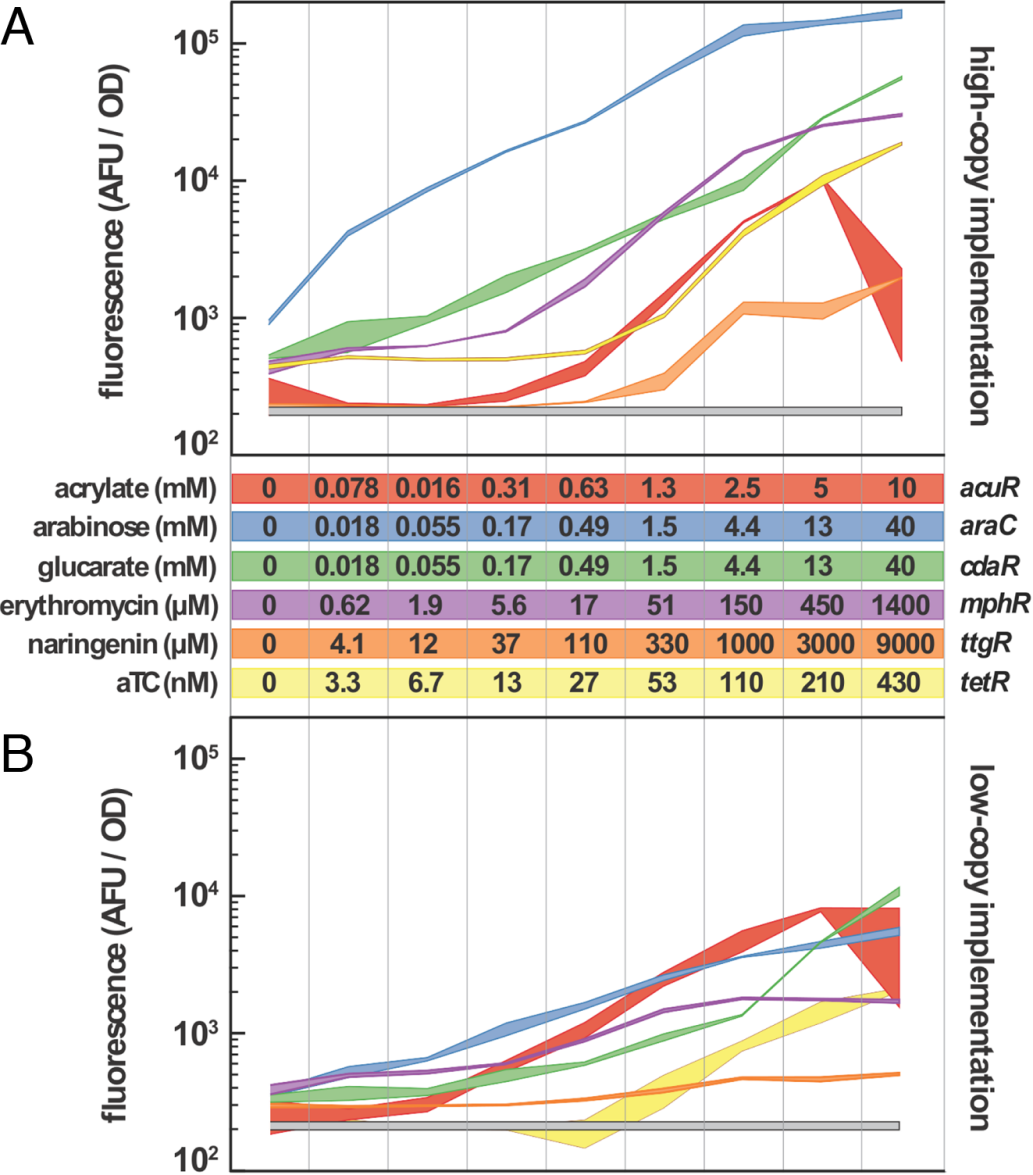
Induction dynamics for each of the inducible systems are reported. The relationship between fluorescent response and inducer concentration is represented as a 95% confidence band (*n* = 3) for both the high-copy (**A**) and low-copy (**B**) implementations of the inducible systems. The plots are log scale to capture the wide range of inducer concentrations and biosensor responses. Inducer concentrations are the same for both high and low-copy implementations. Each curve is matched to a color-coded table of inducer concentration ranges. Acrylate and anhydrotetracycline (aTC) increase in 2-fold increments while arabinose, glucarate, erythromycin and naringenin increase in 3-fold increments. The inducing chemical and biosensor name are indicated to the left and right of the table, respectively. The gray band is the fluorescent response of a control strain containing no fluorescent reporter. Fluorescence measurements are performed 15 h after addition of the inducing chemicals.

**Figure 2.**
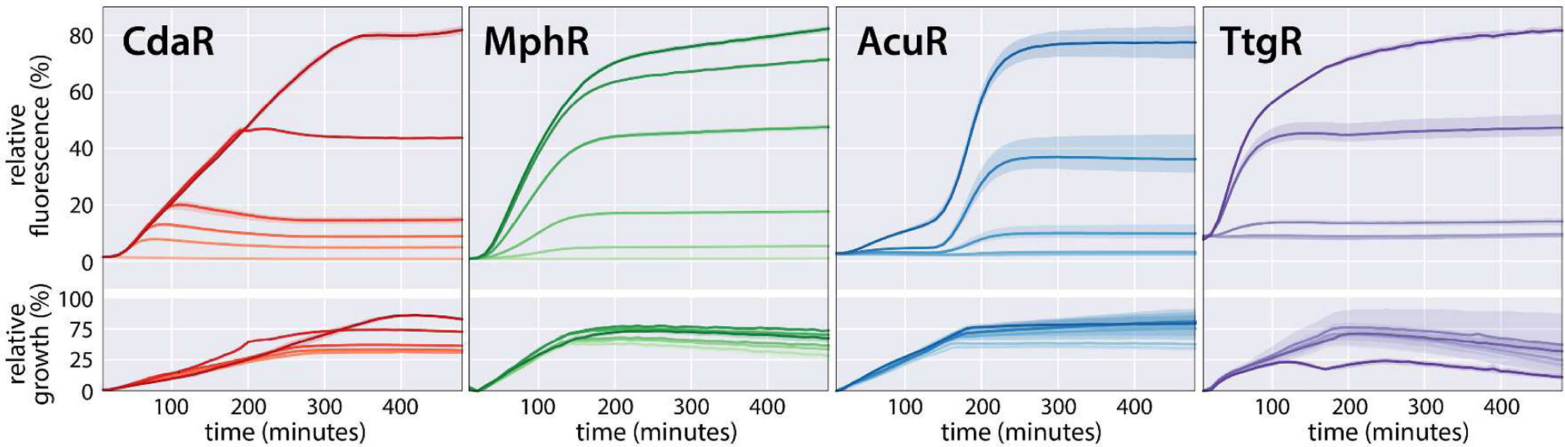
Induction and growth kinetics for the high-copy glucarate (CdaR), erythromycin (MphR), acrylate (AcuR) and naringenin (TtgR) biosensors. Chemical inducers are added at time zero and fluorescence is observed for eight hours. Lower panels show the optical density of the induced cultures over time. Induction levels are indicated by shade, with darker colors indicating higher inducer concentrations. Glucarate induction levels are 40, 13, 4.4, 1.5, 0.49 mM and no inducer addition. Erythromycin induction levels are 1400, 450, 150, 51, 17 μM and no inducer addition. Acrylate induction levels are 5, 2.5, 1.3, 0.63, 0.31 mM and no inducer addition. Naringenin induction levels are 9, 3, 0.33, 0.11, 0.037 mM and no inducer addition. Fluorescence and optical density are normalized as described in the ‘Materials and Methods’ section. The standard error of the mean is represented with a 95% confidence interval (*n* = 3).

The ratio of fluorescence to absorbance at 600 nm was used in order to compensate for changes in cell density over time and between experiments (AFU/OD). Normalized fluorescence at the 15th hour was used to determine the relationship between inducer concentration and fluorescent response. This transfer function is plotted on a log-log scale in Figure 1 to capture the wide range of inducer concentrations and resulting fluorescence values. The Mathematica Hypothesis Testing Package function MeanCI was used to calculate the 95% confidence interval of the estimated mean based upon a Student's *t*-distribution derived from the three induction replicates. Time-courses of cell growth and biosensor activation were normalized and plotted with the Python module Seaborn using bootstrapping to produce 95% confidence intervals for the standard error of the sample mean (assuming a normal error distribution) for the three independently induced replicates (Figure 2, Supplementary Figures S1 and S2). For visualization purposes, normalization was performed on the fluorescence time-course data by dividing all data in a graph by 110% of the highest value such that the trends in each graph can be plotted on a common axis. Growth time-course data was normalized such that each growth curve was divided by its mid-point value and offset to zero at time zero to facilitate co-visualization. The raw data produced by all kinetic induction experiments is provided in Supplementary Material.

Induction ratios were determined after 15 h of induction. Standard error of the derived fold-induction value was determined from the standard error of the mean (assuming a normal error distribution) for the growth-normalized induced, uninduced and control sample means. The quadratic sums of the errors of the values being subtracted were nested within the quadratic sum of the fractional uncertainties of the terms being divided. The resulting equation for the standard error of fold-induction is:

\begin{equation*} \sigma _{\bar F} = \bar F\sqrt {\frac{{\sigma _{\bar I}^2 + \sigma _{\bar C}^2 }}{{\bar I_B^2 }} + \frac{{\sigma _{\bar U}^2 + \sigma _{\bar C}^2 }}{{\bar U_B^2 }}} \end{equation*}

$\bar F$ is the mean magnitude of the fold induction, $\sigma _{\bar I}$, $\sigma _{\bar U}$ and $\sigma _{\bar C}$are the SEM for the fluorescence of induced, uninduced and control cells, while $\bar I_B$ and $\bar U_B$ are the mean fluorescence of the induced and uninduced cells with mean background fluorescence subtracted. For cases where the mean fluorescence of the uninduced cells was within the background fluorescence of the strain, a lower bound on the fold-induction was determined by dividing the 95% confidence interval lower bound of $\bar I_B$ by the 95% confidence interval upper bound of $\bar U_B$. The range of the 95% confidence interval was approximated by doubling the standard error of the background-subtracted fluorescence.

Toxicity of the inducer chemicals and their solvents were determined at each of the concentrations evaluated for induction response. In these experiments, DH5α cells were diluted 1:100 from overnight growth into fresh selective LB and grown for 2 h at 200RPM and 37°C. pUC19 was used as a control plasmid in each case except for the erythromycin evaluation in which pJKR-H-MphR was used to provide *eryR* expression. 150 μl of cells were transferred into 96-well plates and the assayed chemical was immediately added in triplicate before further incubation at 600 RPM and 37°C. After 15 h the absorbance at 600 nm was measured and normalized to the absorbance observed in the control wells in which no chemical was added the cells.

The cross-reactivity matrix was determined by inducing cells that contained target and off-target biosensors. The cells were prepared and evaluated in the same way as described in the induction evaluation experiments. The following inducer concentrations were used: acrylate (5 mM), arabinose (165 μM), glucarate (4.4 mM), erythromycin (51 μM), naringenin (9 mM), IPTG (1 mM), rhamnose (10 mM), cumate (20 μM), DMSO (1%), ethanol (1%).

### Mathematical modeling

The GFP expression rate, normalized for cell growth, was calculated at each time, *t*, with the formula $\Delta GFP_{n,t} = GFP_t /OD_t - GFP_{t - 1} /OD_{t - 1}$. The maximum observed rate of gene expression after addition of inducer was used in subsequent calculations. Scipy was used to perform a non-linear least-squares fit of the maximum GFP expression rate to the corresponding inducer concentration using the Hill function:

\begin{equation*} \left( {\frac{{\Delta GFP_n }}{{\Delta t}}} \right)_{max} = V_{max} \cdot \frac{{I^h }}{{I^h + K_L^h }} + V_{min} \end{equation*}

**Figure 3.**
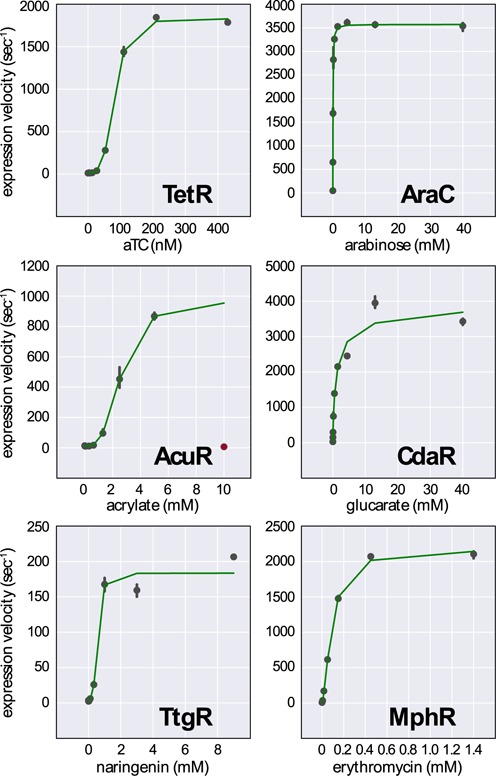
High-copy promoter activity was fit to a model of inducible gene expression. The maximum expression velocity of each inducible promoter was determined at various levels of induction (points). The data was fit to a Hill function modified to account for basal and maximal promoter activity (green lines). The anhydrotetracycline (TetR), acrylate (AcuR) and naringenin (TtgR) biosensors all show high induction cooperativity. The arabinose (AraC), glucarate (CdaR) and erythromycin (MphR) biosensors show low or moderate levels of cooperativity. The 10 mM acrylate induction condition was omitted from the modeling data due to high toxicity (red point). Error bars reflect the 95% confidence interval for the measured expression velocity.

*V_max_* is the maximum rate of GFP expression, *V_min_* is basal rate of GFP expression, *I* is the concentration of inducer, *h* is the hill coefficient and *K_L_* is the lumped half-maximal parameter described in the ‘Results’ section. The variance of each parameter was determined from the least-squares covariance matrix. The square of the variance is the parameter error reported in Table 1. Points in Figure 3 and Supplementary Figure S3 reflect the mean of three independently induced replicates with error bars corresponding to the 95% confidence interval determined for the standard error of the mean by bootstrapping. Lines reflect the model fitted to the data.

**Table 1.** Induction characteristics of the small-molecule inducible systems

	Copy number	Fold induction	Hill coefficient	Half maximal parameter		Max expression velocity (s^−1^)	Basal expression velocity (s^−1^)
AcuR	High	>90	3.2 ± 0.3	2.6 ± 0.1	mM	910 ± 40	5 ± 7
	Low	>50	1.3 ± 0.1	1.0 ± 0.1	mM	150 ± 10	15 ± 2
AraC	High	210 ± 9	1.3 ± 0.1	59 ± 3	μM	3150 ± 60	20 ± 50
	Low	29 ± 3	1.3 ± 0.2	250 ± 30	μM	1260 ± 50	40 ± 30
CdaR	High	168 ± 6	1 ± 0.1	490 ± 60	μM	2600 ± 100	0 ± 60
	Low	78 ± 8	1 ± 0.2	8 ± 2	mM	1000 ± 100	30 ± 20
MphR	High	108 ± 9	1.6 ± 0	97 ± 2	μM	2070 ± 20	9 ± 7
	Low	8 ± 1	1.6 ± 0.1	22 ± 1	μM	66 ± 1	5 ± 0.4
TetR	High	63 ± 3	4.2 ± 0.1	81 ± 1	nM	1760 ± 10	8 ± 6
	Low	>50	3.1 ± 0.3	54 ± 2	nM	116 ± 2	2 ± 1
TtgR	High	70 ± 20	3.8 ± 0.6	550 ± 50	μM	180 ± 6	4 ± 3
	Low	3 ± 0	2.3 ± 0.4	190 ± 20	μM	25 ± 1	6 ± 1

### Flow cytometry

D5Hα cells containing the plasmid to be evaluated were grown to saturation overnight and diluted 1:100 in 1 ml of selective LB media and incubated in 96-well deep well blocks at 900 RPM and 37°C. After 4 h, inducers were added to the desired final concentration and incubation was resumed for 15 h. Induced cultures were diluted 1:100 in cold phosphate buffered saline (PBS) and kept on ice until evaluated on the LSRFortessa flow cytometer (BD Biosciences, San Jose, CA, USA). At least 100 000 events were captured for each sample. Gating was performed on forward and side scatter to avoid debris and clumped cells. Data was exported to FloJo for visualization and Mathematica for subsequent analysis.

Cells that were transformed with pJKR-H-CdaR, MphR-p15a-SPEC-mCherry (designated pJKR-O-MphR) and AcuR-colA-KAN-CFP (designated pJKR-O-AcuR) were maintained in LB with all three antibiotics. The cells were induced in the same manner as above with induction concentrations of 5 mM acrylate, 40 mM glucarate and 37 μg/ml erythromycin. Collection and gating was performed as above. Ten thousand events were plotted in Figure 7 for clarity.

### Glucarate production

For observation of glucarate production via fluorescence, BL21 DE3 (New England Biolabs, Ipswich, MA, USA) cells that were triply transformed with pJKR-H-CdaR, pJKR-UDH and pJKR-MIOX were diluted 1:100 from saturated culture into carbenicillin, spectinomycin and kanamycin selective LB. After 4 h, the cells were transferred to 96-well plates in triplicate and 50 mM myo-inositol was added to the media. Fluorescence and absorbance (600 nm) were measured with a Biotek HT plate reader in 15-min intervals for 48 h with fast shaking at 37°C.

In order to directly measure glucarate titers, BL21 DE3 cells transformed with pJKR-MIOX variants were prepared as above, except for production took place in 1 ml cultures within 96-well deep well blocks incubating at 900 RPM and 37°C for 48 h. Supernatants were collected via centrifugation and filtration and glucarate was determined by mass spectrometry

## RESULTS

We chose to characterize biosensors based upon two criteria: (i) published experimental validation of the DNA binding protein, its cognate promoter/operator and the inducer chemical; and (ii) potential for the inducing chemical to be produced enzymatically through metabolic engineering. AcuR binds acrylate in order to regulate dimethylsulfoniopropionate (DMSP) catabolism in *Rhodobacter sphaeroides* (51). CdaR is a transcriptional activator from *E. coli* that has been shown to regulate transcription in response to several diacids: glucarate, galactarate and glycerate (52). MphR mediates transcription in the presence of erythromycin and other macrolide antibiotics such as josamycin and azithromycin (53). MphR was first identified in a macrolide resistant strain of *E. coli* (53) and has subsequently been used in both mammalian (54,55) and microbial (56) transgene activation. In *Pseudomonas putida*, TtgR regulates expression of a multi-drug efflux pump in response to flavonoids such as naringenin, phloretin and genistein (57), and has also been used for mammalian transgene activation (58). AcuR, MphR and TtgR are members of the TetR transcriptional repressor family. We also included the well-characterized regulators TetR and AraC for comparison.

### Sensor characterization

Biosensors were constructed as a single plasmid encoding both the allosteric transcriptional regulator and a fluorescent reporter. The reporter mRNA is transcribed from a promoter/operator sequence controlled by the allosteric transcriptional regulator. For transcriptional repressors, a medium-strength constitutive promoter (50) was used to drive regulator transcription. For the transcriptional activators, the native promoter sequence of the activator was used in order to preserve the auto-regulating behavior of the AraC and CdaR regulators (52,59). We constructed the biosensors in commonly used high and low copy plasmids to evaluate their behavior in different contexts. High copy plasmids employed a pUC origin of replication (∼100–500 copies), while the low copy plasmids encoded the SC101 replication origin (2–5 copies). All plasmids expressed beta-lactamase, enabling the use of carbenicillin for plasmid maintenance. In the case of the MphR biosensor, an erythromycin resistance gene was also included to protect the cells from the high macrolide concentrations required for induction.

The relationship between inducer concentration and expression of the fluorescent reporter was evaluated for six inducible systems (Figure 1). The resulting biosensor transfer functions encompass the complete range of sensor outputs, allowing determination of each biosensor's dynamic range. Evaluation of the transfer function also reveals the minimum and maximum expression level obtainable in each biosensor implementation. The calculated fold-induction (maximum fluorescence divided by uninduced fluorescence) of high-copy biosensors ranged from 63 to 210 (Table 1). The fold-induction values indicated as being above a certain number are the result of the mean uninduced fluorescence residing within, or very near, the intensity of the cellular auto-fluorescence. A true number for fold-induction is undefined in this scenario, so a minimum fold-induction was determined using the bounds of the 95% confidence intervals. The greatest magnitude of induction among the high-copy biosensors was achieved with AraC, followed by CdaR and MphR (Figure 1a). For the low-copy biosensors, fold induction ranged from 3 to 78. The AcuR biosensor demonstrated the lowest uninduced accumulation of GFP, with no fluorescence above background in the absence of acrylate for both the high and low copy systems. This is in contrast to the TtgR biosensor, which showed higher uninduced accumulation of GFP in the low-copy configuration. The opposite effect was observed for the TetR biosensor, which demonstrated a lower uninduced accumulation of GFP, such that fluorescence was within that of the background in the low-copy configuration (Figure 1b).

The time required for induction was evaluated for each biosensor (Figure 2, Supplementary Figures S1 and S2). Reporter expression was monitored for eight hours with a wide range of inducer concentrations. All high-copy biosensors began producing fluorescence above background within 30 min, and achieved maximum levels of fluorescence within 5 h under the highest induction conditions. Low-copy biosensors began producing measurable fluorescence within 50 min, but could require >8 h to achieve maximum fluorescence at the highest levels of induction. While the onset of expression began rapidly and without much variability between biosensors, the maximum fluorescence was sensor-dependent. For example, the CdaR biosensor achieved maximum fluorescence from moderate induction in nearly 1 h, while the highest glucarate induction condition required 6 h to reach maximal fluorescence. This is in contrast to the high-copy MphR biosensor, which approached maximal fluorescence around three hours regardless of the intensity of induction. The low copy variant of the MphR biosensor showed a similar trend, but required additional time to achieve maximum fluorescence (Supplementary Figure S1). Variability in the kinetics of induction may be related to the intrinsic strength of the regulated promoter, sensor-DNA equilibrium, or the relationship between biosensor activity and growth-phase.

We observe several types of biosensor induction behavior. Figure 2 reveals that the MphR and TtgR biosensors have similar modes of induction with initial rates of GFP accumulation proportional to the intensity of induction. In contrast, the CdaR biosensor shows more similar rates of GFP accumulation for a range of inducer concentrations, with higher concentrations of glucarate resulting in longer periods of GFP accumulation. This behavior may be the result of the auto-regulatory interaction between CdaR and the CdaR promoter. An intermediate mode of fluorescence accumulation is observed for the TetR biosensors (Supplementary Figure S2), with the high-copy variant showing similar initial rates of GFP accumulation for the two highest induction levels and the low-copy variant showing more varied initial rates of GFP accumulation. With the exception of AcuR, repressor-mediated biosensors cease to accumulate additional fluorescence at the onset of stationary phase. This is in contrast to the activators, which achieve maximal fluorescence well before stationary phase. Contrary to the behavior of the other repressors, induction of AcuR by acrylate results in substantial GFP accumulation after entry into stationary phase.

Complex synthetic circuits can be mathematically modeled to aid in component selection and system design. In order to make our biosensors compatible with such forward engineering efforts, we applied a simple model of gene activation to relate promoter activity to inducer concentration. We defined promoter activity as the time derivative of fluorescence corrected for cell growth. The time required for fluorophore maturation was considered and found to be less than two minutes (49). Likewise, degradation of GFP was ignored because the half-life in *E. coli* is >24 h (60). We fit gene expression rates to a Hill function adapted to account for both the maximum velocity of gene expression and the basal expression of the uninduced cells. We found that both activators, AraC and CdaR, had Hill coefficients indicating low cooperatively. The repressors TetR, AcuR and TtgR all exhibited high cooperatively. The exception is the repressor MphR, which has a lower Hill coefficient (Table 1). Examination of the activity-induction curves of the high-copy sensors reveals that the induction behavior of MphR is indeed more similar to that of the activators AraC and CdaR, rather than the other repressors TetR, AcuR and TtgR (Figure 3). The same trend holds for the activity-induction curves of the low-copy sensors except that AcuR demonstrates less cooperatively in this implementation, potentially due to its dependence on growth phase for activation (Supplementary Figure S3). Due to the toxicity of acrylate at 10 mM, we omitted this induction condition from the data used for modeling. Likewise, the highest concentrations of erythromycin were omitted from the low-copy MphR biosensor model as they showed substantial toxicity, likely due to lower expression of the erythromycin resistance gene. The maximum velocity of the high-copy sensors was always greater than the low-copy versions, however the magnitude of the change was greater in the repressors than the activators. The activator-based sensors control their own expression and this feedback may provide some expression stability in the face of copy number variation. Basal promoter activity was less than 3% of the maximum promoter activity for each high-copy biosensor. Low-copy biosensors had higher and more variable basal promoter activity due to lower maximum activities, and in some cases, less effective transcriptional repression.

**Figure 4.**
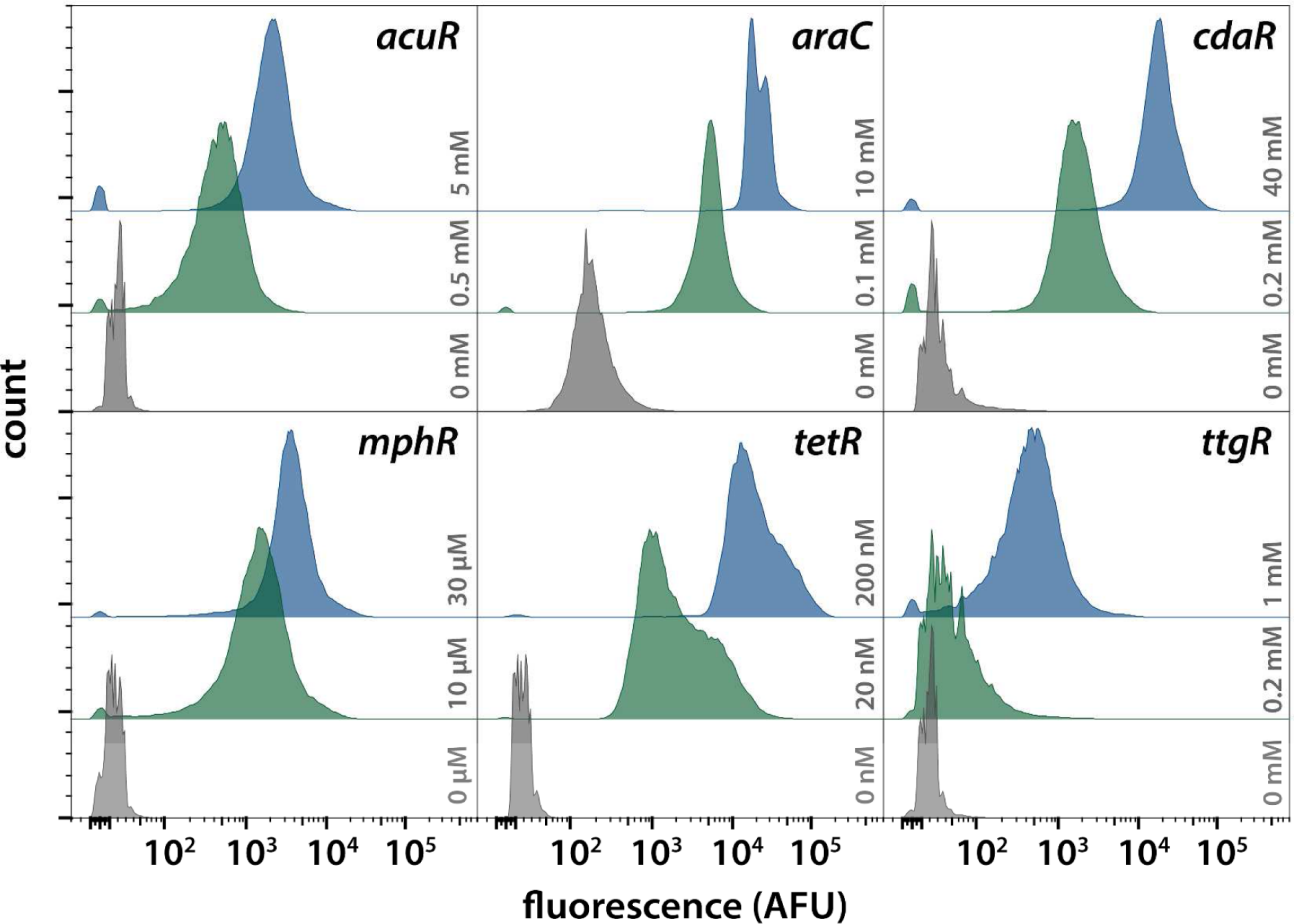
The behavior of single cells in response to chemical induction was evaluated by flow cytometry. 100 000 cells from uninduced (gray), partially induced (green) and fully induced (blue) populations were observed for each high copy biosensor. The specific concentration of inducer is indicated in the plot. Histograms are plotted with a biexponential scale to render the wide range of biosensor activation. The absence of large, well-separated bimodal distributions indicates that bulk fluorescent measurements do indeed reflect the induction behavior of individual cells.

Individual cells were evaluated by flow cytometry to determine whether the ensemble induction dynamics were indicative of single cell behavior, or were instead an averaged result of stochastic, all-or-nothing responses in individual cells (Figure 4, Supplementary Figure S4). This type of characterization is important, as some inducible systems have been observed to produce bimodal or otherwise heterogeneous induction patterns due to positive feedback or inducer transport properties (61,62). Basal, low and high induction levels were measured after overnight induction. For each biosensor, it was shown that the majority of individual cells adjust their fluorescence in response to inducer concentration. In cases where a small group of cells do not fluoresce, the population represents less than 2% of the total cell population and may consist of dead cells, or cells containing dysfunctional plasmids. Nonetheless, the individual cell responses reflect the population-averaged behavior observed in ensemble measurements. High-copy AraC and CdaR biosensors both have high basal levels of reporter expression when evaluated in bulk (Figure 1a). Unsurprisingly, these sensors demonstrated the widest uninduced fluorescence distributions when evaluated at the single cell level (Figure 4). The TetR biosensor has a broad fluorescence distribution when partially induced, possibly precluding its use in sensitive induction applications. When partially induced, both low copy MphR and high copy TtgR fluorescence distributions are compressed against the limit of detection (Supplementary Figure S4). This could indicate that the left tail of the distribution is below the limit of detection, or that some cells are not activating in response to the inducer. As observed in the ensemble measurements, TtgR induction is weak. In the case of the low copy TtgR plasmid, the induced and uninduced populations almost entirely overlap when observed by flow cytometry (Supplementary Figure S4).

**Figure 5.**
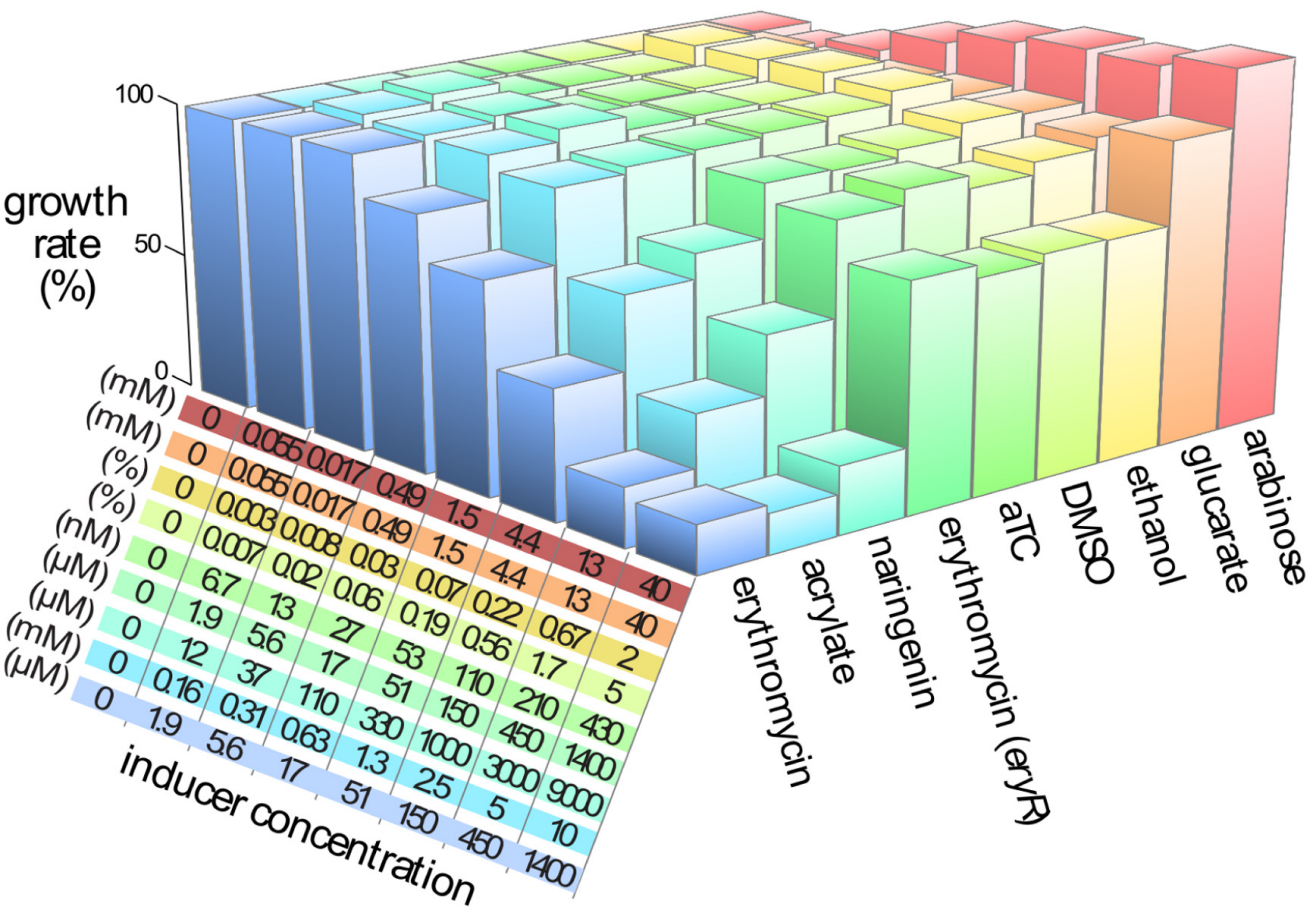
The toxicity of each inducer chemical was evaluated over a wide range of concentrations. Growth rate was measured for each combination of chemical and concentration during the exponential phase of growth. Rates were normalized to the growth rate of cells without any added chemical and plotted as bar height. Concentration of each inducer is indicated in the table, corresponding to the bar chart by order and color. Ethanol and DMSO were included as they are the solvents for aTC and naringenin, respectively. Erythromycin was evaluated twice: with and without the erythromycin resistance gene, *eryR*. Inducer concentrations mirror the concentrations used in the induction experiments.

Toxicity of the inducer chemicals was measured to help guide the choice of inducer concentration for future biosensor applications (Figure 5). In applications where maximum protein production is the goal, toxicity will be less of a consideration. In contrast, sub-toxic induction is important for complex circuits that require the cell to maintain a healthy cell state. As expected, erythromycin was toxic to *E. coli* at concentrations as low as 50 μM. However, with expression of the erythromycin resistance gene (*eryR*), only slight toxicity was observed at erythromycin concentrations up to 1.4 mM. A similar growth defect was observed with 430 nM anhydrotetracycline (aTC). Both growth defects are likely due to the solvent, in this case ethanol. Naringenin showed significant toxicity at concentrations of 330 μM and above. This toxicity is likely due to the flavonoid itself, rather than the solvent dimethylsulfoxide (DMSO). Acrylate showed substantial toxicity at 5 mM and 10 mM, corroborating previous observations (63). High concentrations of arabinose resulted in higher growth rates due to *E. coli's* ability to use the sugar as a carbon source. Similar but more modest growth benefits were observed at the highest concentration of glucarate and at low levels of ethanol supplementation.

### Sensor orthogonality

**Figure 6.**
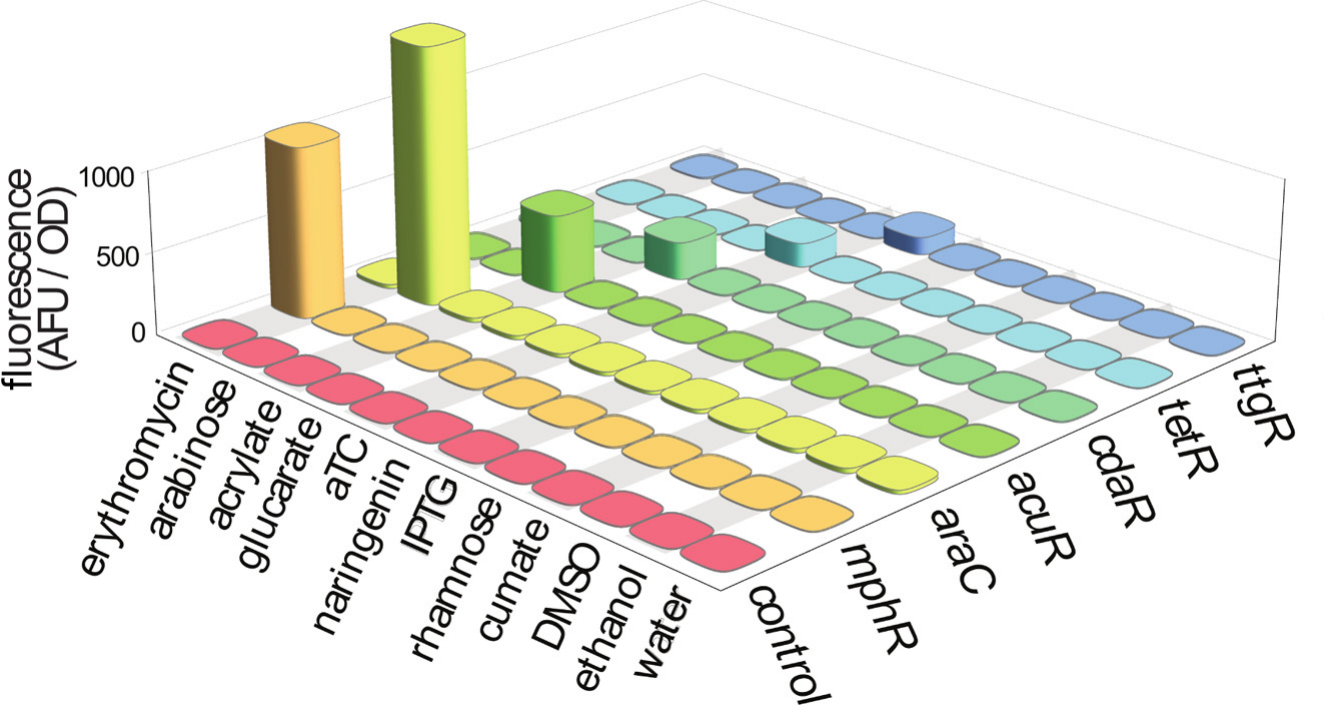
The potential for the chemical inducers to activate non-target sensors was evaluated. The cross-reactivity of the new inducers, along with a selection of other commonly used inducers and inducer solvents, was evaluated against each of our six inducible systems. The growth-normalized fluorescent response of each biosensor-inducer pair is plotted as height (*n* = 3). No cross-reactivity was observed.

The cross-reactivity of each biosensor was evaluated with a panel of inducing compounds: acrylate, arabinose, glucarate, erythromycin, aTC, naringenin, IPTG, rhamnose, cumate and the solvents, DMSO and ethanol. The inducing compounds not otherwise evaluated in this work were included to provide forward compatibility for future biosensor implementations. No sensor was observed to respond to any of the evaluated compounds except for its cognate inducer (Figure 6). Cumate, glucarate and acrylate all feature a carboxylate, yet are discriminated by their respective sensors. TtgR is known to be promiscuous (57,64), and it is surprising that it is not activated by cumate since it binds many similar molecules, one of which is chloramphenicol (57). TtgR activation by chloramphenicol precludes engineered systems containing TtgR alongside a plasmid maintained by chloramphenicol acetyl transferase.

**Figure 7.**
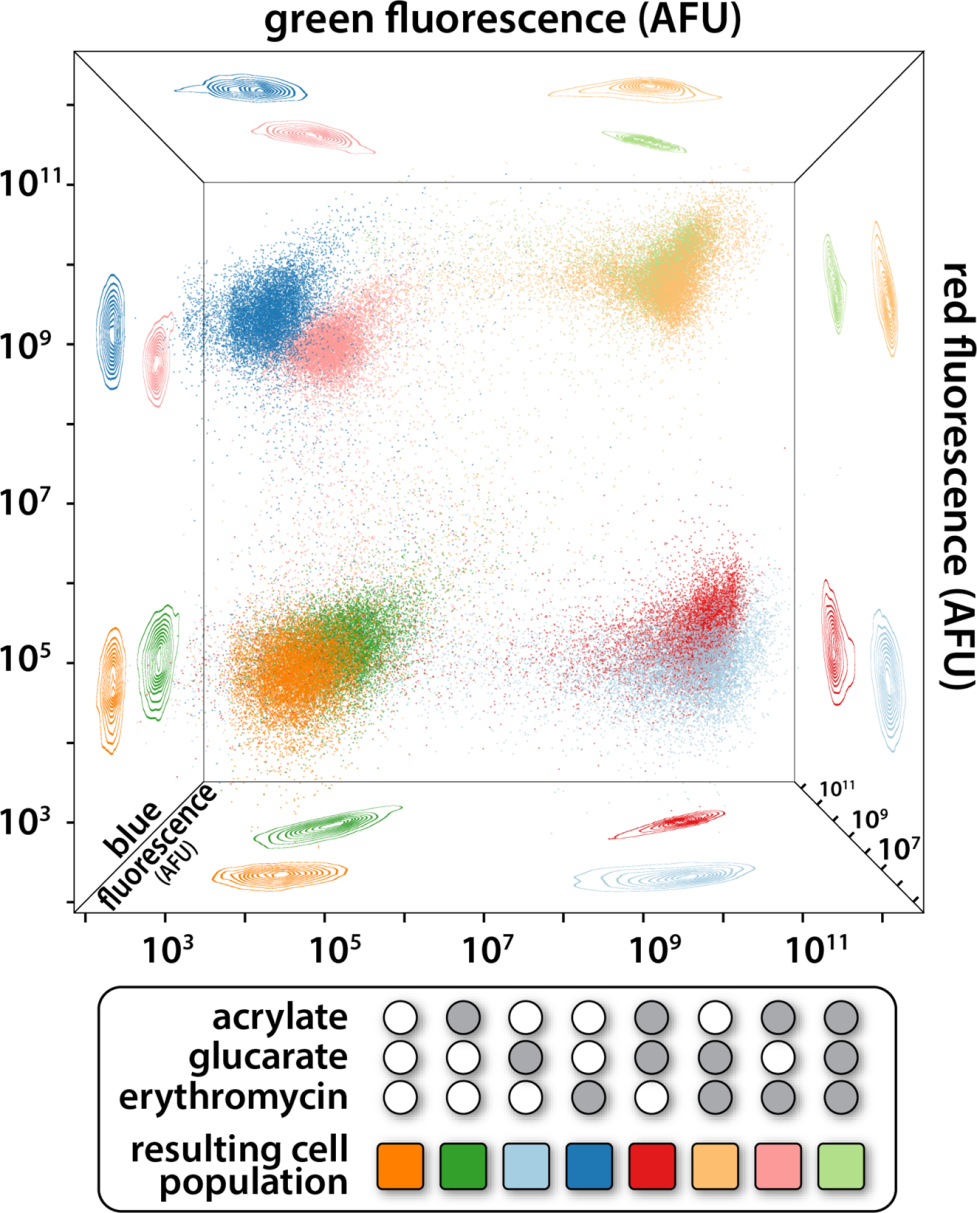
Compatible CdaR-GFP, AcuR-CFP and MphR-mCherry biosensors were transformed into the same cell. The potential for these biosensors to be controlled independently was evaluated by flow cytometry. The isogenic cell population was exposed to no inducer (orange), glucarate (light blue), acrylate (dark green), erythromycin (dark blue), glucarate and acrylate (red), glucarate and erythromycin (tan), erythromycin and acrylate (pink) or glucarate, acrylate and erythromycin (light green). The eight combinations of binary induction resulted in eight distinct cell populations when characterized in the three fluorescent channels. The point clouds, each point representing 1 of 10 000 cells, are projected onto the faces of the cube in order to aid in visualization of the 3D space. All axes are log scale to capture the wide range of fluorescent responses.

While the cross-reactivities of the biosensors were evaluated with a single sensor per cell, the real utility of orthogonal sensors comes from controlling a single cell with multiple sensors. To this end, we redeployed several sensors to allow stable maintenance and non-overlapping fluorescent readouts in the same cell. The MphR biosensor was reconstructed such that erythromycin controlled the expression of mCherry in a vector backbone encoding the p15a replication origin and spectinomycin resistance. Similarly, the AcuR biosensor was reconstructed in a vector backbone encoding the colA origin and kanamycin resistance to facilitate acrylate-mediated expression of CFP. These plasmids were co-transformed with the pJKR-H-CdaR plasmid (encoding GFP) and stably maintained in DH5α cells. Overnight induction of this strain with every combination of glucarate, erythromycin and acrylate induction resulted in eight distinct cell states as measured by fluorescence in the three channels (Figure 7). High, but non-toxic, levels of inducer were chosen for each orthogonal induction channel.

Flow cytometry was used to evaluate individual cell behavior. Without induction, there is low fluorescence in all channels, as represented by the orange population in Figure 7. Induction with only glucarate results in individual cells changing their cell state by producing GFP with no CFP or RFP expression (light blue population in Figure 7). The trend continues with induction by erythromycin producing the dark blue cell population exhibiting high fluorescence in the red channel, but low fluorescence in the blue and green channels. Similarly, the dark green points represent acrylate-induced cells with high fluorescence in the blue channel. Induction by all three ligands results in the light green cell population demonstrating high fluorescence in all three channels.

In principle, 16 distinct cell states can be defined with four orthogonal inducible systems, and 32 distinct cell states can be defined with five inducible systems. In these cases, output channels become limiting, as there are limited distinct fluorescent proteins. If three levels of induction are considered (none, intermediate and high), rather than the binary case examined here, the number of cell states increases from 8 to 27 for the system of three orthogonal biosensors, and from 16 to 81 for the theoretical system of four orthogonal biosensors.

### Sensors for metabolic flux monitoring

**Figure 8.**
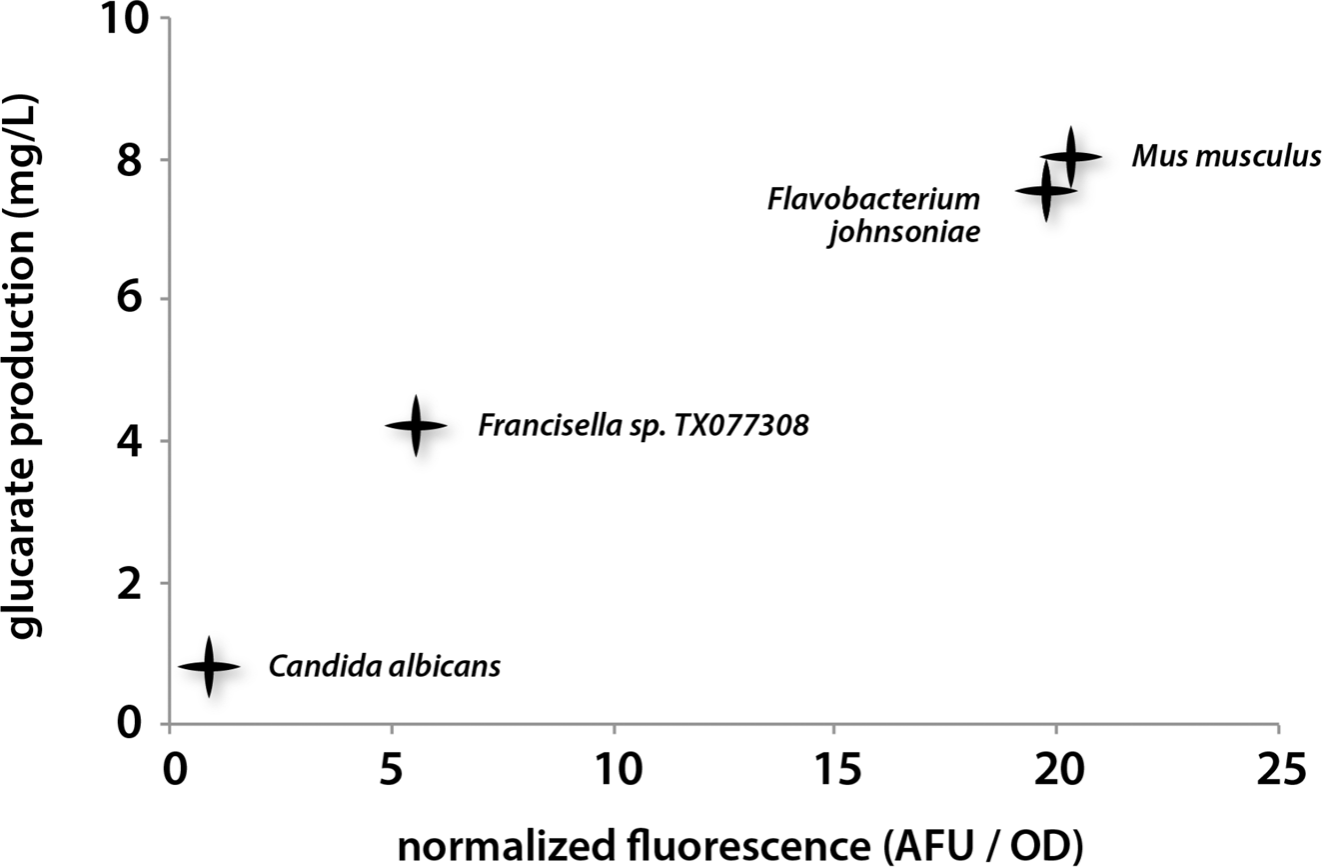
Activation of the CdaR biosensor is well correlated with glucarate titers. Glucarate can be produced from myo-inositol by the enzymes MIOX and Udh. MIOX orthologs were transformed into cells containing Udh and the CdaR biosensor. Fluorescence was observed 48 h after addition of myo-inositol. Glucarate titers were measured after the same period of time in identical strains without the glucarate biosensor. All coefficients of variation are less than 10% (*n* = 3).

The CdaR biosensor was used to monitor production of glucarate from myo-inositol. Glucarate can be produced from biomass as a renewable replacement for nylon and other plastics (65), however high titers are currently limited by the activity of myo-inositol oxygenase (MIOX) (66), which converts myo-inositol into glucuronate. Glucuronate is in turn oxidized to glucarate by the fast-acting enzyme glucuronate dehydrogenase (Udh). By co-transforming plasmids containing the CdaR biosensor, a constitutively expressed Udh gene and a library of four constitutively expressed MIOX orthologs, we were able to rapidly identify enzymes producing higher glucarate titers in *E. coli*. The four MIOX variants produced a 20-fold range in fluorescence after 16 h (Figure 8). Mass spectrometry was used to determine actual glucarate titers in order to determine if biosensor readout was predictive of an enzyme's potential for glucarate production. Glucarate titers were well correlated with fluorescence (Figure 8), encouraging future work in which biosensors enable high throughput discovery and optimization of enzymatic activity. The previously characterized *Mus musculus* MIOX ortholog (66,67) produced the highest fluorescence and titer. Interestingly, a very similar glucarate titer and biosensor response was obtained from the *Flavobacterium johnsoniae* MIOX ortholog that shares only 45% identity with the *M. musculus* variant.

**Figure 9.**
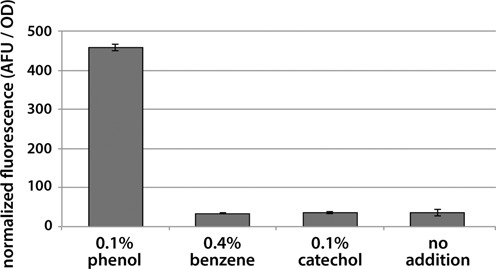
The TtgR biosensor was evaluated for its ability to aid in the directed evolution of enzymatic phenol production. A fluorescent response was observed in the presence of 0.1% phenol, while background levels of fluorescence were observed when the sensor was induced with the precursor molecule benzene. Catechol is a side-product of phenol production and did not activate the sensor. All experiments were carried out in triplicate.

Phenol is an important commodity chemical for which novel production routes would provide economic and environmental benefits. Some success has been shown in enzymatic conversion of benzene to phenol (68,69), however these enzymes function with low *k*_cat_, and in some cases continue oxidizing phenol to catechol, an undesirable side product (69). We hypothesized that TtgR would be able to respond to phenol as it responds to many other compounds with an activated benzene. Separate populations of the TtgR biosensor strain were incubated with 0.1% phenol, 0.1% catechol and up to 0.4% benzene. Surprisingly, only phenol activated the sensor (Figure 9). The selective response of TtgR to phenol, but not byproducts of phenol production, may enable the directed evolution of phenol-producing enzymes by coupling phenol production to expression of fluorescent proteins or antibiotic selection markers in individual cells.

## DISCUSSION

Enabling the simultaneous control of multiple reporter-coupled genes expands our ability to rapidly probe cellular behavior. Characterization of new and interoperable genetically encoded sensors provides additional input and output nodes for engineered biological systems. Demonstrating the utility of these sensors in metabolic engineering applications provides a basis for future work in directed evolution and enzyme discovery applications.

A balance in the basal strength of the regulated promoter, the copy number of that promoter, and the expression level of the regulator protein is key in achieving tight repression with high dynamic range upon induction. Such behavior has been exploited in previously well-characterized inducible systems to achieve tuned responses to induction (70–72). The TtgR biosensor is an example of a promoter/repressor system that would benefit from further tuning of these parameters. The low dynamic range of the TtgR biosensor and the observation that shifting from a high- to low-copy sensor implementation results in elevated basal reporter expression may be characteristics of a poorly tuned system. Future work will involve multiplexed evaluation of these design specifications in order to understand how system characteristics such as wide dynamic range, low off-state expression and ultra-high expression can be achieved.

By applying a modeling framework to our biosensors, we are able to capture their behavior with a small number of key parameters. Basal and maximal promoter activities have clear biological meaning while the Hill coefficient and lumped half-maximal parameter are more complicated. For inducible systems regulated by a transcription factor, the lumped half-maximal parameter of the Hill equation represents $K \cdot K_d \cdot K_p \cdot T$, where *K* is an equilibrium constant reflecting the binding event(s) between the transcription factor and the promoter, *K_d_* is the disassociation constant for inducer bound to transcription factor, *K_p_* is the partition coefficient for intracellular to extracellular inducer, and *T* is the concentration of transcription factor in the cell (73). The *K_d_* is known for several of the transcription factors evaluated here, however the partitioning coefficient and equilibrium of the DNA-protein binding events are not. We did not determine the absolute concentration of transcription factor in the cells, however we note that the lumped parameter is almost always smaller in the low-copy biosensor variants, reflecting the lower relative concentration of transcription factor. We fit the half-maximal parameter as a lumped value, as we are unable to deconvolute the individual parameters that make it up.

The magnitude of the Hill coefficient reflects the cooperatively of the system, with higher values resulting in more sigmoidal induction curves. The Hill coefficient determined for both low- and high-copy AraC is 1.3, which is in close agreement with previously measured values that range between 1 and 2 (74). The Hill coefficients for low- and high-copy TetR are 3.1 and 4.2, which are similar to previously reported values (75,76). The TtgR biosensor has low- and high-copy Hill coefficients of 2.3 and 3.8, respectively. The CdaR biosensors demonstrated no cooperatively with a Hill coefficient of 1. The lack of cooperatively may be the result of CdaR acting as a monomer, or because there is feedback between biosensor activation and CdaR expression. The moderate Hill coefficient of 1.6 observed for the MphR biosensors reflects a cooperativity substantially lower than the other high-copy repressors. As noted above, the AcuR biosensor shows substantially different induction responses in the high- and low–copy implementations, possibly due to its dependence on growth-phase for induction.

Demonstration of biosensor interoperability is critical for complicated synthetic biological applications in which multiple genes need to be controlled simultaneously (77). We approached interoperability characterization in two ways: by demonstrating that only cognate inducer compounds activated a given sensor (Figure 6) and by combining three biosensors, each controlling an orthogonal output, in a single cell (Figure 7). Ideally, we would have constructed a cell containing every biosensor; however, spectral overlap between fluorescent reporter proteins constrained the system. Reporter protein expression was similar in strength for singly, doubly and triply induced populations for each of the three biosensors evaluated: AcuR, MphR and CdaR. This result confirms our expectation that cellular behavior prototyped with a single sensor should maintain a similar behavior when operated alongside at least two other inducible systems. While the cell states defined in Figure 7 are discussed as a digital system, in reality, induction level is a continuous variable and analog descriptions of system dynamics are more precise (78).

Finally, we demonstrated the use of CdaR and TtgR as biosensors in the observation of intracellular metabolite concentrations. In the case of CdaR, we were able to demonstrate that more effective variants of the MIOX enzyme provided a higher fluorescent signature when the production phenotype was observed through biosensor response. Using fluorescence as a proxy for metabolite production will enable screening millions of enzyme variants per day, rather than the thousands of metabolites that can be screened by relying solely on HPLC or mass spectrometry (24). The TtgR biosensor was found to respond to phenol, a valuable compound that was not previously known to bind and activate the TtgR transcriptional repressor. Crucial for the directed evolution of phenol producing enzymes is the observation that TtgR does not respond to the precursor molecule, benzene, or the unwanted byproduct, catechol.

Innovation in the characterization of biological parts is enabling new options for biological design. Fluorescent activated cell sorting (FACS) combined with multiplexed DNA synthesis and sequencing has been used to characterize biological parts at a throughput of more than 10,000 parts per experiment (79). While these parts were constitutive promoter and RBS variants, advances in microfluidics have enabled new strategies for the characterization of inducible systems. These strategies allow culture conditions to be changed over time while maintaining cells in specific growth phases (80). Combined with automated microscopy and image processing, these microfluidic platforms open the door for more comprehensive characterization pipelines (81).

Thorough characterization of biological parts is the bedrock for abstraction and complexity in engineered biological systems. Of primary importance are sensors, as they provide channels of communication into and out of the cell. *A priori* design of complex biological systems is challenging, and greater success may be achieved by first engineering a simple reporter that monitors the desired phenotype (*e.g*. metabolite production). Once the desired phenotype produces an output such as fluorescence, millions if not billions of designs can be evaluated rapidly, allowing biological engineering to more closely mimic the process of evolution for which the biological medium was optimized.

## SUPPLEMENTARY DATA

Supplementary Data are available at NAR Online.

SUPPLEMENTARY DATA
