# Supplementary material for: Synthetic biosensors for precise gene control and real-time monitoring of metabolites
Source: Nucleic Acids Res. 2015 Jul 7;43(15):7648–60. doi: 10.1093/nar/gkv616 (PMC4551912; doi:10.1093/nar/gkv616)
Supplement: SUPPLEMENTARY DATA [file supp_43_15_7648__index.html]

Synthetic biosensors for precise gene control and real-time monitoring of metabolites — Synthetic biosensors for precise gene control and real-time monitoring of metabolites — SUPPLEMENTARY DATA 

# Synthetic biosensors for precise gene control and real-time monitoring of metabolites

## SUPPLEMENTARY DATA

- SUPPLEMENTARY DATA
- SUPPLEMENTARY DATA
