# Supplementary material for: Synthetic biosensors for precise gene control and real-time monitoring of metabolites
Source: Nucleic Acids Res. 2015 Jul 7;43(15):7648–60. doi: 10.1093/nar/gkv616 (PMC4551912; doi:10.1093/nar/gkv616)
Supplement: SUPPLEMENTARY DATA [file supp_gkv616_nar-03524-y-2014-File011.pdf]

Synthetic biosensors for precise gene control and real-time monitoring of metabolites.

Jameson K. Rogers[1,2], Christopher D. Guzman[1], Noah D. Taylor[1,3], Srivatsan Raman[1,3], Kelley Anderson[1] and George M. Church[1,3,*]

[1] Wyss Institute for Biologically Inspired Engineering, Harvard University, 3 Blackfan Circle, Boston, MA – 02115, USA

[2] School of Engineering and Applied Sciences, Harvard University, 29 Oxford Street, Cambridge, MA – 02143, USA

[3] Department of Genetics, Harvard Medical School, 77 Avenue Louis Pasteur, Boston, MA – 02115, USA

[*] To whom correspondence should be addressed. E-mail: gchurch@genetics.med.harvard.edu

Supplemental Figure 1
Fluorescence and growth kinetics for the low-copy implementations of the glucarate, erythromycin, acrylate and and naringenin biosensors.

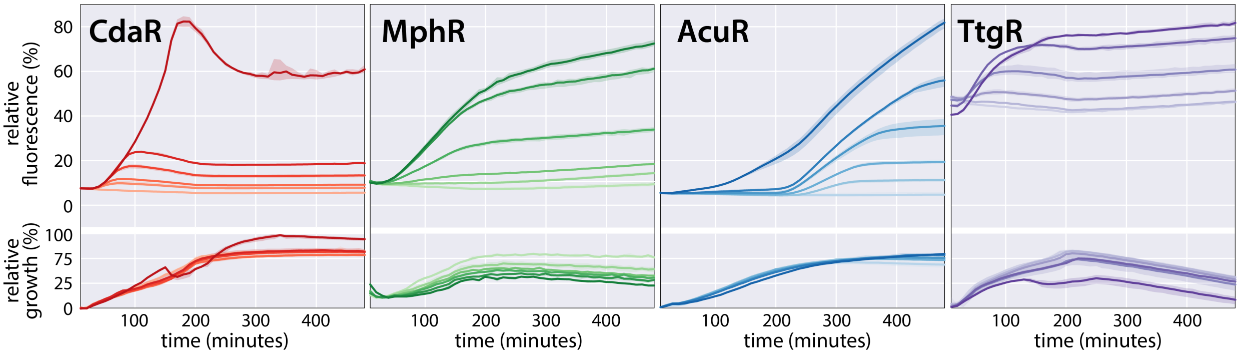

Induction and growth kinetics for the low-copy glucarate (CdaR), erythromycin (MphR), acrylate (AcuR) and naringenin (TtgR) biosensors. Chemical inducers are added at time zero and fluorescence is observed for eight hours. Lower panels show the optical density of the induced cultures over time. Induction levels are indicated by shade, with darker colors indicating higher inducer concentrations. Glucarate induction levels are 13mM, 4.4mM, 1.5mM, 0.49mM, 0.17mM and no inducer addition. Erythromycin induction levels are 150µM, 51µM, 17µM, 5.6µM, 1.9µM and no inducer addition. Acrylate induction levels are 5mM, 2.5mM, 1.3mM, 0.63mM, 0.31mM and no inducer addition. Naringenin induction levels are 9mM, 3mM, 0.33mM, 0.11mM, 0.037mM and no inducer addition. Fluorescence and optical density are normalized as described in the Methods. The standard error of the mean is represented with a 95% confidence interval (n=3).

Supplemental Figure 2
Fluorescence and growth kinetics for the arabinose and anhydrotetracycline (aTC) biosensors.

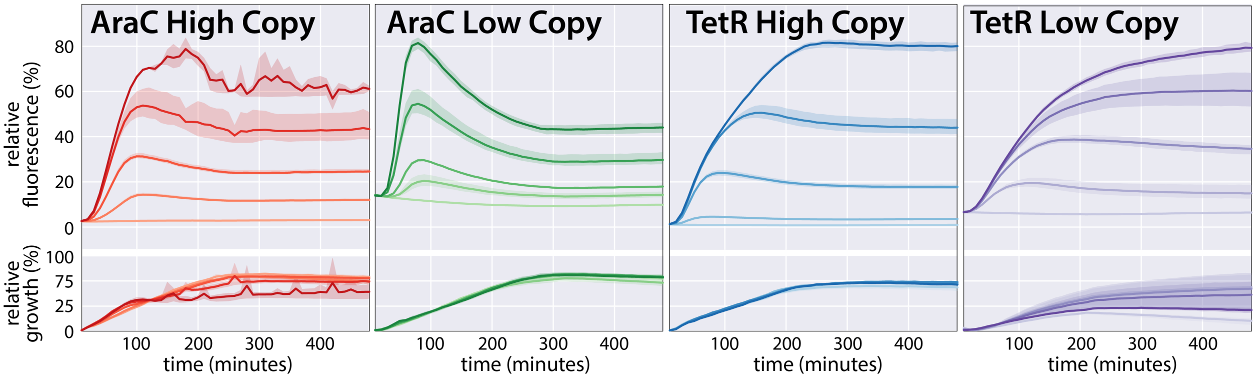

Induction and growth kinetics for the high- and low-copy arabinose (AraC) and anhydrotetracycline (TetR) biosensors. Chemical inducers are added at time zero and fluorescence is observed for eight hours. Lower panels show the optical density of the induced cultures over time. Induction levels are indicated by shade, with darker colors indicating higher inducer concentrations. Arabinose induction levels are 490µM, 170µM, 55µM, 18µM and no inducer addition. Anhydrotetracycline induction levels are 430nM, 210nM, 110nM, 53nM and no inducer addition. Fluorescence and optical density are normalized as described in the Methods. The standard error of the mean is represented with a 95% confidence interval (n=3).

Supplemental Figure 3
Promoter activities and model fits for the low-copy biosensors.

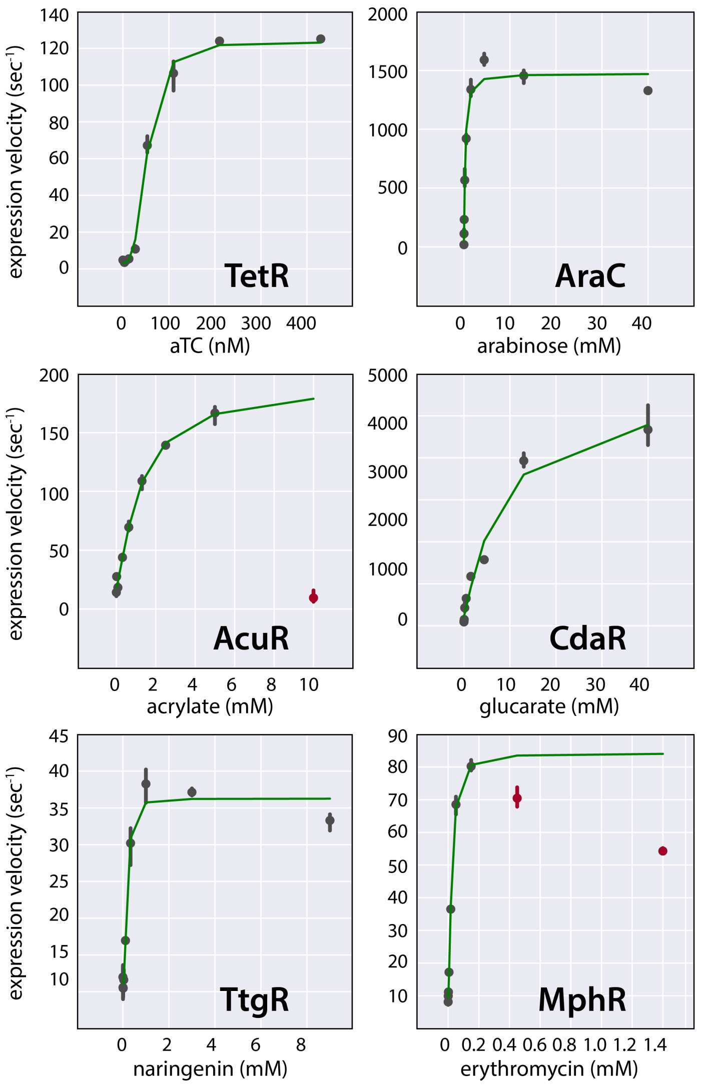

Low-copy promoter activity was fit to a model of inducible gene expression. The maximum expression velocity of each inducible promoter was determined at various levels of induction (points). The data was fit to a Hill function modified to account for basal and maximal promoter activity (green lines). The anhydrotetracycline (TetR) and naringenin (TtgR) biosensors show high induction cooperativity. The arabinose (AraC), glucarate (CdaR), acrylate (AcuR) and erythromycin (MphR) biosensors show low or moderate levels of cooperativity. The 10mM acrylate, 1400µM and 450µM erythromycin induction conditions were omitted from the modeling data due to high toxicity (red points). Error bars reflect the 95% confidence interval for the measured expression velocity.

Supplemental Figure 4
Single cell analysis of the low-copy biosensors.

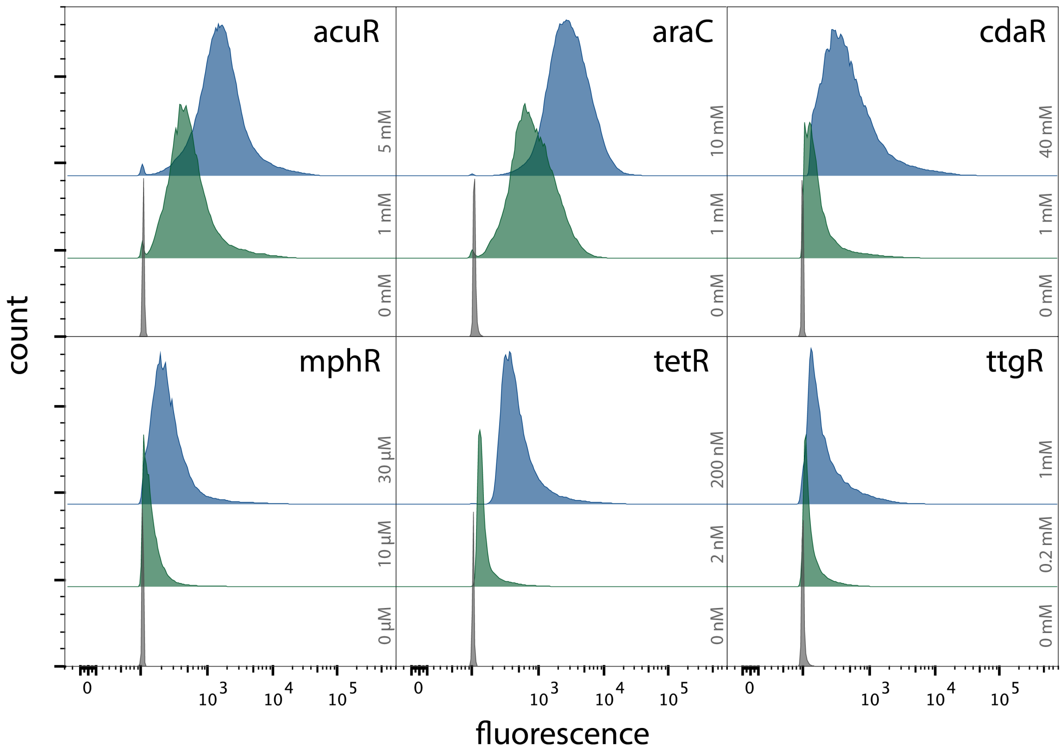

The behavior of single cells in response to chemical induction was evaluated by flow cytometry. 100,000 cells from uninduced (grey), partially induced (green) and fully induced (blue) populations were observed for each low-copy biosensor. The specific concentration of inducer is indicated in the plot. Histograms are plotted with a biexponential scale to render the wide range of biosensor activation. The absence of large, well-separated bimodal distributions indicates that bulk fluorescent measurements do indeed reflect the induction behavior of individual cells.

Supplemental Table 1. Sequence of regulator proteins and cognate promoter/operators.

Regulator	Promoter / Operator Sequence	Regulator Sequence
acuR	GCTTCACAACCGCACTTGATTTAATAGA<br>CCATACCGTCTATTATTTCTGG	ATGCCGCTGACCGACACCCCGCCGTCTGTTCCGCAGAAACCGCGTCGTGGTCGTCCGCGTGGTGCTCCG<br>GACGCTTCTCTGGCTCACCAGTCTCTGATCCGTGCTGGTCTGGAACACCTGACCGAAAAAGGTTACTCTT<br>CTGTTGGTGTTGACGAAATCCTGAAAGCTGCTCGTGTTCCGAAAGGTTCTTTCTACCACTACTTCCGTAAC<br>AAAGCTGACTTCGGTCTGGCTCTGATCGAAGCTTACGACACCTACTTCGCTCGTCTCCTCGACCAGGCGT<br>TCCTGGACGGTTCGCTGGCTCCGCTGGCTCGTCTGCGTCTGTTCACCCGTATGGCTGAAGAAGGTATGGC<br>TCGTCACGGTTTCCGTCGTGGTTGCCTGGTTGGTAACCTGGGTCAGGAAATGGGTGCTCTGCCGGACGA<br>CTTCCGTGCTGCTCTGATCGGTGTTCTGGAAACCTGGCAGCGTCGTACCGCTCAGCTGTTCCGTGAAGCT<br>CAGGCTTGCGGTGAACTGTCTGCTGACCACGACCCGGACGCTCTGGCTGAAGCTTTCTGGATCGGTTGG<br>GAAGGTGCTATCCTGCGTGCTAAACTGGAACTGCGTCCGGACCCGCTGCACTCTTTCACCCGTACCTTCG<br>GTCGTCACTTCGTTACCCGTACCCAGGAATAA
araC	AGAAACCAATTGTCCATATTGCATCAGA<br>CATTGCCGTCACTGCGTCTTTTACTGGCT<br>CTTCTCGCTAACCCAACCGGTAACCCCG<br>CTTATTAAAAGCATTCTGTAACAAAGCG<br>GGACCAAAGCCATGACAAAAACGCGTA<br>ACAAAAGTGTCTATAATCACGGCAGAAA<br>AGTCCACATTGATTATTTGCACGGCGTC<br>ACACTTTGCTATGCCATAGCATTTTTATC<br>CATAAGATTAGCGGATCCTACCTGACGC<br>TTTTTATCGCAACTCTCTACTGTTTCTCCA<br>TACCCGCTTTCATATCTTTCACTTTTTTTG<br>GGCTAAC	ATGGCTGAAGCGCAAAATGATCCCCTGCTGCCGGGATACTCGTTTAACGCCCATCTGGTGGCGGGTTTA<br>ACGCCGATTGAGGCCAACGGTTATCTCGATTTTTTTATCGACCGACCGCTGGGAATGAAAGGTTATATTC<br>TCAATCTCACCATTCGCGGTCAGGGGGTGGTGAAAAATCAGGGACGAGAATTTGTCTGCCGACCGGGTG<br>ATATTTTGCTGTTCCCGCCAGGAGAGATTCATCACTACGGTCGTCATCCGGAGGCTCGCGAATGGTATCA<br>CCAGTGGGTTTACTTTCGTCCGCGCGCCTACTGGCATGAATGGCTTAACTGGCCGTCAATATTTGCCAAT<br>ACGGGTTTCTTTCGCCCGGATGAAGCGCACCAGCCGCATTTCAGCGACCTGTTTGGGCAAATCATTAACG<br>CCGGGCAAGGGGAAGGGCGCTATTCGGAGCTGCTGGCGATAAATCTGCTTGAGCAATTGTTACTGCGG<br>CGCATGGAAGCGATTAACGAGTCGCTCCATCCACCGATGGATAATCGGGTACGCGAGGCTTGTCAGTAC<br>ATCAGCGATCACCTGGCAGACAGCAATTTTGATATCGCCAGCGTCGCACAGCATGTTTGCTTGTCGCCGT<br>CGCGTCTGTCACATCTTTTCCGCCAGCAGTTAGGGATTAGCGTCTTAAGCTGGCGCGAGGACCAACGCAT<br>TAGTCAGGCGAAGCTGCTTTTGAGCACTACCCGGATGCCTATCGCCACCGTCGGTCGCAATGTTGGTTTT<br>GACGATCAACTCTATTTCTCGCGAGTATTTAAAAAATGCACCGGGGCCAGCCCGAGCGAGTTTCGTGCC<br>GGTTGTGAAGAAAAAGTGAATGATGTAGCCGTCAAGTTGTCAtaa
cdaR	ATGCTGTTGATTGACGCCAGTGAGAACC<br>CGGAACCGGAAACGGAATCAAATCCGT<br>GGGTCGAACAGTGGGGCACGCTGTTGT<br>CCTGATATGTTCAGCGAGCGGTAAATGT<br>CGTTTTAGCGGTGCTGAATCGAATCTTT<br>TCAGGCAAATGCCAGTAAAAACTGCTTC<br>ATAGCGCGGATTTTTACTGGCGTTTGCC<br>TGGAGTCAAGCGATCCATTTCATACTCTT<br>CTTTATTTCTTCGTTTTAACCCTTCCTTTC<br>TTGTTCTTGTTTTCATTTCCGTGAAGTGG<br>ATTCCACCGTCCAGGGCTAATGCCAAAA<br>TCGGGCCTCATTGAACGCATTAATGTTG<br>TGTTGTTGCACGGTGAGCCGCTATGGCG<br>CGCTTTTTATACTGCTATTGCCAGATATA<br>AACACGCGCCGTATTCGGCGAACGACCT<br>ATAAAAACGGCAAAAAACACCCTACGTC<br>ACCTCTGATTTCCTGGCGATGTCGCAGT<br>CCAGAGTGAGCGTGGCTAACGCGAATT<br>TTCAGGAGTGCAACA	ATGGCTGGCTGGCATCTTGATACCAAAATGGCGCAGGATATCGTGGCACGTACCATGCGCATCATCGAT<br>ACCAATATCAACGTAATGGATGCCCGTGGGCGAATTATCGGCAGCGGCGATCGTGAGCGTATTGGTGAA<br>TTGCACGAAGGTGCATTGCTGGTACTTTCACAGGGACGAGTCGTCGATATCGATGACGCGGTAGCACGT<br>CATCTGCACGGTGTGCGGCAGGGGATTAATCTACCGTTACGGCTGGAAGGTGAAATTGTCGGCGTAATT<br>GGCCTGACAGGTGAACCAGAGAATCTGCGTAAATATGGCGAACTGGTCTGCATGACGGCTGAAATGAT<br>GCTGGAACAGTCGCGGTTGATGCACTTGTTGGCGCAGGATAGCCGTTTGCGGGAAGAACTGGTGATGA<br>ACCTGATTCAGGCAGAGGAGAATACTCCCGCACTTACTGAATGGGCGCAACGGCTGGGGATCGATCTCA<br>ATCAACCGCGAGTGGTGGCTATTGTTGAGGTCGACAGCGGTCAGCTTGGCGTGGACAGCGCAATGGCG<br>GAGTTACAACAACTGCAAAACGCGCTGACTACGCCCGAGCGTAATAATCTGGTGGCGATTGTCTCGCTA<br>ACCGAAATGGTGGTGTTGAAACCGGCGTTGAACTCTTTTGGGCGCTGGGATGCAGAAGATCATCGTAAG<br>CGAGTTGAACAACTGATTACCCGCATGAAAGAGTACGGCCAGCTGCGTTTTCGCGTTTCACTGGGCAACT<br>ATTTTACCGGTCCTGGCAGTATTGCCCGATCCTATCGTACGGCGAAAACGACGATGGTGGTGGGTAAAC<br>AGCGGATGCCAGAAAGTCGCTGCTATTTTTATCAGGATCTGATGTTACCTGTGTTACTCGACAGTTTGCG<br>TGGCGACTGGCAGGCCAACGAACTGGCGCGACCGCTGGCGCGGCTGAAAACGATGGACAATAACGGCT<br>TGCTGCGACGAACGCTGGCGGCGTGGTTTCGCCACAATGTGCAACCGCTGGCAACGTCAAAGGCGTTGT<br>TTATTCATCGTAATACCCTGGAGTATCGGCTTAATCGTATATCGGAACTGACCGGGCTTGATTTGGGCAA<br>TTTTGATGACAGGTTGCTGCTGTATGTGGCGTTACAACTGGATGAAGAGCGGtag
mphR	GGATTGAATATAACCGACGTGACTGTTA<br>CATTTAGGTGGCTAAACCCGTCAA	ATGCCGCGTCCGAAACTGAAATCTGACGACGAAGTTCTGGAAGCGGCGACCGTTGTTCTGAAACGTTGC<br>GGTCCGATCGAATTCACCCTGTCTGGTGTTGCGAAAGAAGTTGGTCTGTCTCGTGCGGCGCTGATCCAG<br>CGTTTCACCAACCGTGACACCCTGCTGGTTCGTATGATGGAACGTGGTGTTGAACAGGTTCGTCACTACC<br>TGAACGCGATCCCGATCGGTGCGGGTCCGCAGGGTCTGTGGGAATTCCTGCAGGTTCTGGTTCGTTCTA<br>TGAACACCCGTAACGACTTCTCTGTTAACTACCTGATCTCTTGGTACGAACTGCAGGTTCCGGAACTGCG<br>TACCCTGGCGATCCAGCGTAACCGTGCGGTTGTTGAAGGTATCCGTAAACGTCTGCCGCCGGGTGCGCC<br>GGCGGCGGCGGAACTGCTGCTGCACTCTGTTATCGCGGGTGCGACCATGCAGTGGGCGGTTGACCCGG<br>ACGGTGAACTGGCGGACCACGTTCTGGCGCAGATCGCGGCGATCCTGTGCCTGATGTTCCCGGAACACG<br>ACGACTTCCAGCTGCTGCAGGCGCACGCGTAA
tetR	TCGAGTCCCTATCAGTGATAGAGATTGA<br>CATCCCTATCAGTGATAGAGATACTGAG<br>CACATCAGCAGGACGCACTGACCGAATT<br>CATTAAA	ATGTCTCGTTTAGATAAAAGTAAAGTGATTAACAGCGCATTAGAGCTGCTTAATGAGGTCGGAATCGAA<br>GGTTTAACAACCCGTAAACTCGCCCAGAAGCTAGGTGTAGAGCAGCCTACATTGTATTGGCATGTAAAA<br>AATAAGCGGGCTTTGCTCGACGCCTTAGCCATTGAGATGTTAGATAGGCACCATACTCACTTTTGCCCTTT<br>AGAAGGGGAAAGCTGGCAAGATTTTTTACGTAATAACGCTAAAAGTTTTAGATGTGCTTTACTAAGTCAT<br>CGCGATGGAGCAAAAGTACATTTAGGTACACGGCCTACAGAAAAACAGTATGAAACTCTCGAAAATCAA<br>TTAGCCTTTTTATGCCAACAAGGTTTTTCACTAGAGAATGCATTATATGCACTCAGCGCAGTGGGGCATTT<br>TACTTTAGGTTGCGTATTGGAAGATCAAGAGCATCAAGTCGCTAAAGAAGAAAGGGAAACACCTACTAC<br>TGATAGTATGCCGCCATTATTACGACAAGCTATCGAATTATTTGATCACCAAGGTGCAGAGCCAGCCTTC<br>TTATTCGGCCTTGAATTGATCATATGCGGATTAGAAAAACAACTTAAATGTGAAAGTGGGTCTTAA
ttgR	CACCCAGCAGTATTTACAAACAACCATG<br>AATGTAAGTATATTCCTTAGCAA	ATGGTGCGTCGCACCAAAGAAGAAGCACAGGAAACGCGTGCGCAGATTATCGAAGCGGCCGAACGCGC<br>GTTTTATAAACGTGGTGTGGCACGTACCACGCTGGCAGATATTGCAGAACTGGCAGGTGTTACCCGCGG<br>TGCAATCTACTGGCATTTCAACAATAAAGCCGAACTGGTTCAGGCACTGCTGGATTCTCTGCACGAAACG<br>CATGATCACCTGGCCCGTGCAAGCGAATCTGAAGATGAACTGGACCCGCTGGGCTGCATGCGCAAACTG<br>CTGCTGCAGGTGTTTAACGAACTGGTTCTGGATGCACGTACCCGTCGCATTAATGAAATCCTGCATCACA<br>AATGCGAATTTACGGATGATATGTGTGAAATTCGTCAGCAGCGCCAGAGCGCCGTGCTGGATTGTCATA<br>AAGGTATCACCCTGGCACTGGCAAACGCAGTTCGTCGCGGTCAGCTGCCGGGTGAACTGGATGTGGAA<br>CGCGCAGCGGTTGCGATGTTTGCCTATGTGGATGGCCTGATTGGTCGTTGGCTGCTGCTGCCGGATAGT<br>GTTGATCTGCTGGGCGATGTGGAAAAATGGGTTGATACCGGTCTGGATATGCTGCGTCTGAGCCCGGCG<br>CTGCGCAAATAA

Supplemental Table 2. Sequence of MIOX orthologs evaluated in this study.

MIOX Variant	Sequence
Candida albicans	ATGGTAAACAAGGTCGGTAAATCTACTCTCGATAAGAGCACAAACCTAGATAAATCCAAAGGGAATATATTAGA<br>GAAACTAGATGATGATATACTTCATGTCAATAGAATTCGAGGCTCTTTAACTAACAAAACTCCAATCACCAAAACC<br>CATTCGATAGATGATGAGCTTAAACTAGAAGAACAATCAGAAACTGCCGCCGATGAAAATTGGCAAATAGCATC<br>GGAATATTATAAAAACATAGACACGAAGGCTTTCCGCCAATATGAATTAGCTTGTGATAGAGTCAAACAGTTTTA<br>TGAAGAACAACATGAAAAACAAACCGTGGCGTATAATATTCAAGCAAGAATTAATTTCAAAACTAAAACAAGAG<br>CAAGAATGACAGTTTGGGAAGGACTAGAGAAATTAAACAAATTGTTAGATGATTCTGATCCCGACACCGAATTGT<br>CACAAATAGATCATGCATTACAGACGGCAGAAGCTATACGGCGAGATGGGAAACCACGATGGTTTCAATTAGTT<br>GGGTTGATTCATGATTTAGGGAAATTACTATATTTTTTTGATTCTCGTGGTCAATGGGATGTAGTGGGTGATACTT<br>TCCCTGTTGGTTGTAAATTCCTGAAACGGATTATTTTCCCTGATAGTTTTAAAAATAATCCAGATTTCCTAAATCCA<br>TTGTATAATACCAAATATGGCATATATTCAAAACATTGTGGATTAGATAAAGTCATGTTGAGTTGGGGTCATGAT<br>GAGTATATGTATCATGTTGCGAAAAAGAATTCGACATTACCACCGGAAGCATTGGCAATGATAAGGTATCATTCA<br>TTTTATCCTTGGCATCAAGAATTGGCATATAGTTATTTAATGGATGAGCATGATAAAGAGATGTTGAAAGCAGTC<br>AAAGCTTTCAATTCCTATGATTTATATTCCAAGATAGATCAACAGTATGATGTTGAAGAGTTGAAACCATATTACC<br>TAGAGTTGATTGATGAGTTTTTCCCAAATAAAGTAATTGATTTTTAA
Francisella sp. TX077308	ATGAGTCAGACCGTGGAAAACACGTTTGGCGAATTTCGTAACTACACCGATAGCAAATTCCAGGATCGTGTGGA<br>ACGCACGTACAAAGATATGCACATTAACCAGAATCTGGAATACGTTACCCAGATGAAAGATAAATACTTCAAACT<br>GGATCTGGGTAAAATGGATGTGTACGAAGTTTTCAAACTGCTGGAAAACGTTCATGATGAAAGCGATCCGGATA<br>ATGATCTGCCGCAGATCGAACACGCATATCAGACCGCGGAAGCCTGCCAGAACAAATTCCTGAAATCTGATACG<br>GAACTGCGCGAAAATGCGCTGATTCGTAGTATCTTTCGCGATCATGAATGGCAGAGCATTCCGAAAATCTGGCAG<br>GATTTCTATACCAAAAAACAGAGTCTGGGCAATCTGTACAGCCATATTAAAGATTGGTCTTGGTTTCCGCTGGTTG<br>GCTTCGTTCACGATCTGGGTAAAATCATGACCCTGCCGGAATATGGTCAGCTGCCGCAGTGGAGCACCGTGGGT<br>GATACGTACCCGATTGCCTGCCCGTTTGCAAGCGCGAACGTGTTTTCTCACCGTGAATTTGTTAAAGATTCTAAAG<br>ATTACAACAATTACAATACCGAAAGTGAAACGCATTATGGCAAATACGAGAAAAAATGTGGTTTCGATAACGTG<br>GATATGAGCTTCGGTCACGATGAATACATCTACAAAGTTTTCGAACAGGGCAGCGATATCCCGTATGAAGGTCTG<br>TACCTGCTGCGCTATCATTCTTTCTACCCGTGGCACACCCCGCAGACGGGCGGTCATGCGTATCAGGAACTGGCC<br>AACGAAAAAGATTGGCTGCTGCTGCCGCTGCTGAAAGCCTTTCAGAAAGCGGATCTGTATTCTAAACTGCCGGAA<br>CTGCCGCCGAAAGAAGTGCTGGAGAAAAAATACAAAAGTCTGCTGGATAAATGGGTTCCGAACAAGAAAATTAA<br>CTGGTAA
Flavobacterium johnsoniae	ATGAAAAAGCATATAGACACAGACAATCCGTTGAAAAATTTAGATGAGTGGGAAGATGATTTGTTAATGCGATA<br>TCCTGACCCTTCTGAAGTAAATGAAAGTTTAAAAGAAAAGCAGAAAGAAGAATTTAGAAATTATGTCGATTCTGA<br>AAGAGTAGAAACGGTAAAAGAATTTTACAGGATAAACCATACCTACCAAACTTATGACTTTGTATGCAGTAAAGA<br>ACAAGAATTTCTGCAATTTAATAGAAAAGAAATGTCAATCTGGGAAGCTGTCGAGTTTTTAAACACGCTTGTAGA<br>CGACAGTGACCCAGATATTGACTTAGACCAGACACAGCACCTTTTACAGACTTCAGAAGCCATTCGTGCTGATGG<br>TCATCCGGATTGGTTTGTACTGACAGGTTTCATTCACGATTTGGGTAAAGTTTTATGCTTATTTGGAGAACCGCAA<br>TGGGCAGTCGTTGGCGATACTTTTCCGGTTGGCTGTGCGTATTCGGATAAAATTGTGTATTCAGAATTTTTTAAAG<br>AAAATCCGGATTATACAGATGAGAGATTCAATACTAAACTAGGAATCTACACTGAAAACTGCGGATTAGATAACG<br>TAAAAATGAGCTGGGGTCATGACGAATATTTGTATCAGATTATGAAAGATTATTTACCGGATCCTGCTTTATACAT<br>GATTCGTTATCACTCTTTTTATTCGCAGCATAAAGAAAATGCGTATGCACATTTAATGAATGAAAAAGACATCGAA<br>ATGTTTGACTGGGTTCGAAAATTCAATCCGTACGATTTGTATACAAAGGCTCCTGTAAAACCAGATGTTCAGGCAT<br>TACTTCCTTATTATAAAGAATTAGTTGCTAAATATTTGCCTGAAAAATTGAAGTTTTAA
Mus musculus	ATGAAAGTGGATGTTGGCCCGGACCCGAGCCTGGTTTACCGCCCGGATGTGGACCCGGAAATGGCAAAAAGCA<br>AAGATTCGTTTCGTAACTACACCAGTGGCCCGCTGCTGGATCGTGTTTTTACCACGTATAAACTGATGCATACCCA<br>CCAGACGGTTGACTTTGTCAGCCGTAAACGCATTCAATATGGCGGTTTCTCTTACAAGAAAATGACCATCATGGA<br>AGCGGTGGGCATGCTGGATGACCTGGTTGATGAATCAGATCCGGACGTCGATTTTCCGAATTCGTTTCATGCGTT<br>CCAGACGGCCGAAGGTATTCGCAAAGCCCACCCGGACAAAGATTGGTTCCATCTGGTCGGCCTGCTGCACGATCT<br>GGGTAAAATCATGGCACTGTGGGGTGAACCGCAGTGGGCTGTGGTTGGTGATACCTTTCCGGTGGGTTGCCGTC<br>CGCAAGCAAGTGTCGTGTTTTGTGACTCCACCTTCCAGGACAACCCGGATCTGCAAGACCCGCGCTATTCAACGG<br>AACTGGGCATGTACCAGCCGCATTGCGGTCTGGAAAACGTGCTGATGTCGTGGGGTCACGATGAATACCTGTAC<br>CAGATGATGAAATTCAACAAATTCAGCCTGCCGTCTGAAGCCTTCTACATGATCCGTTTCCATAGTTTCTACCCGT<br>GGCACACCGGCGGTGATTATCGCCAGCTGTGCTCCCAGCAAGACCTGGATATGCTGCCGTGGGTGCAAGAATTC<br>AACAAATTCGATCTGTACACGAAATGTCCGGATCTGCCGGACGTTGAATCTCTGCGTCCGTACTACCAAGGTCTG<br>ATTGATAAATACTGTCCGGGCACCCTGTCGTGGTAA

Supplemental Table 3. Inducer toxicity.

Inducer Concentration								
**acrylate(μM)**	0	156	313	625	1250	2500	5000	10000
**arabinose (μM)**	0	55	165	494	1481	4444	13333	40000
**aTC (nM)**	0	6.7	13.0	27	53	110	210	430
**DMSO (%)**	0	0.0069	0.021	0.062	0.19	0.56	1.7	5
**erythromycin (no eryR, μM)**	0	1.9	5.6	17	51	150	450	1400
**erythromycin (μM)**	0	1.9	5.6	17	51	150	450	1400
**ethanol (%)**	0	0.0027	0.0082	0.025	0.074	0.22	0.7	2
**glucarate (μM)**	0	55	165	494	1481	4444	13333	40000
**naringenin (μM)**	0	12	37	111	333	1000	3000	9000
Growth Rate (hr^{-1})								
**acrylate**	0.73	0.75	0.74	0.73	0.70	0.50	0.27	0.10
**arabinose**	0.78	0.76	0.80	0.86	0.90	0.92	0.92	0.95
**aTC**	0.74	0.75	0.74	0.75	0.75	0.70	0.70	0.54
**DMSO**	0.73	0.74	0.74	0.74	0.75	0.71	0.66	0.55
**erythromycin (no eryR)**	0.68	0.68	0.67	0.69	0.68	0.65	0.61	0.52
**erythromycin**	0.67	0.67	0.67	0.58	0.48	0.29	0.13	0.11
**ethanol**	0.70	0.75	0.75	0.76	0.75	0.71	0.66	0.52
**glucarate**	0.74	0.74	0.74	0.74	0.74	0.74	0.72	0.76
**naringenin**	0.69	0.73	0.72	0.72	0.68	0.53	0.40	0.16

Supplemental Table 4. Inducer cross-reactivity (growth-normalized fluorescence)

	TtgR	TetR	CdaR	AcuR	AraC	MphR	control
erythromycin	11	9	14	11	25	1063	8
arabinose	8	8	11	10	1609	8	6
acrylate	9	8	9	485	27	10	6
glucarate	7	7	236	10	27	9	7
aTC	8	152	10	10	24	10	8
naringenin	111	7	9	8	24	8	7
IPTG	8	7	9	10	22	10	8
rhamnose	8	8	10	11	25	10	8
cumate	8	8	9	9	22	9	8
DMSO	8	8	9	9	26	10	8
ethanol	8	6	8	8	30	8	6
water	8	6	8	7	24	7	6
